# MicroRNA-300: A Transcellular Mediator in Exosome Regulates Melanoma Progression

**DOI:** 10.3389/fonc.2019.01005

**Published:** 2019-10-15

**Authors:** Long Chen, Vega Windy Karisma, Huawen Liu, Li Zhong

**Affiliations:** ^1^Bioengineering Institute of Chongqing University, Chongqing, China; ^2^Three Gorges Central Hospital, Chongqing, China

**Keywords:** UV, Gadd45b, miR-300, exosome, melanoma

## Abstract

Melanoma is a common and high-mortality skin cancer. Oxidative stress and DNA damage caused by ultraviolet light (UV) are major causative factors of melanoma formation. However, the specific molecular mechanism is still unclear. In this study, 218 dysregulated genes and 104 dysregulated miRNAs in response to UV were screened by analyzing sequencing datasets. Among them, 29 up-regulated miRNAs and 28 down-regulated miRNAs were involved in the melanoma pathway. As the only differential gene in the melanoma pathway, GADD45B severely affects the prognosis of melanoma patients. MiR-300 is the only differentially expressed miRNA that regulates GADD45B. In addition, compared to normal melanocytes, miR-300 was significantly down-regulated in melanoma cells (log FC = −1.63) and exosomes (log FC = −1.34). Among the transcription factors predicted to regulate miR-300, MYC, PPARG, and ZIC2 were significantly up-regulated in melanoma cells, and TP53, JUN, JUNB, FOS, and FOSB interacted with GADD45B. We attempted to reveal the pathogenesis of melanoma and screen new biomarkers by constructing a TF-mRNA-miRNA axis in turn to provide a view for further research.

## Introduction

Melanoma is one type of skin cancer with high incidence, poor prognosis, and a complicated mechanism. UVR (ultraviolet ray) is one of the most important among many oncogenic factors. The greatest source of UVR reaching the earth is sunlight, composed of 95% UVA (320–340 nm) and 5% UVB (290–320 nm), while UVC is blocked by the aerosphere (1, 2). Both UVA and UVB could cause DNA damage and alter the skin microenvironment (3–5). Epidemiological studies suggest potential roles of UVA and UVB in melanoma formation, but the comparative importance of these studies remains controversial. Melanocytes, which are neural crest cell derivatives, produce melanin pigment, and transfer it to adjacent cells like keratinocytes via melanosomes. In the epidermal layer of the skin, melanocytes and surrounding keratinocytes form the melanin units (6). UVR activates signaling cascade that could induce melanin synthesis in melanocytes (7–10).

Skin pigmentation requires close intercellular communication which not only results in suntan but also constitutes a defense mechanism against nuclei UVR damage (11). Thus, intercellular interactions and signal transduction between melanocytes and keratinocytes might play a key role in melanoma progression. Cell communication often occurs via either liposoluble factors or extracellular vesicles (EV) (12, 13). Exosomes are endosome-derived EVs released into the extracellular environment. Exosomes comprise membrane vesicles and cytosolic components such as nucleic acids, proteins and lipids (14, 15). Once in contact with target cells, membrane fusion occurs and releases inside elements. In Alessandra's study, it was demonstrated that exosomes released from keratinocytes play a role in the regulation of pigmentation. Exosomes carrying various selected microRNAs (miRNAs) target melanocytes and modulate the melanogenesis by altering gene expression and enzyme activity (16).

MiRNAs, which are 20–25 nucleotides in length, could target specific mRNAs causing mRNA degradation or blocking translation through RISC (RNA-induced silencing complex) (17). Over the past few years, miRNAs were reported as new regulators of various events in skin physiology. Different miRNAs are implicated in melanogenesis through targeting MITF (microphthalmia-associated transcription factor) or other related genes. Melanogenesis could reduce the risk of cancer via preventing UV to penetrate into melanocytes and inhibiting DNA damage. However, UVB with short wavelengths could trigger oxidative stress. The equilibrium of the skin microenvironment might be broken concomitantly, which then becomes the main cause of melanoma initiation. Thus, studying the molecular mechanisms of preventing oxidative damage prevention is also relevant.

Growth arrest and DNA-inducible gene products, including Gadd45a, Gadd45b, and Gadd45g, are involved in stress signaling in response to endogenous and exogenous stressors. These gene products have been shown be involved with cell cycle arrest, DNA repair, and cell apoptosis (18). For instance, GADD45 has been shown to stimulate the p38-JNK(c-Jun N-terminal kinase)-MAPK (mitogen-activated protein kinase) pathways in response to stress (19–21). In addition, Nrf2 (nuclear factor NF-E2-related factor 2), an important antioxidant element, is also an upstream transcription factor that activates Gadd45b expression against oxidative stress (22). In hepatic oxidative stress, it is also verified that STAT3 ubiquitination and degradation leads to increased expressions of Gadd45b (23). Although Gadd45b is supposed to be linked with melanoma, the mechanism has yet to be clarified. Thus, the relevance of investigating the role of Gadd45b in oxidative stress-induced melanoma oncogenesis.

**Figure 1:**
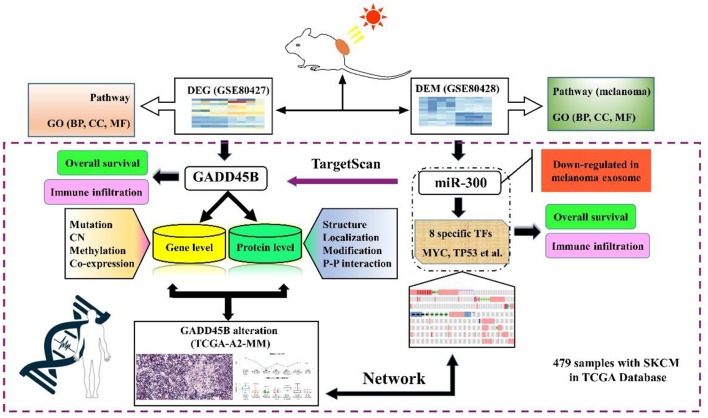
The flow chart of the study.

The causes behind the mechanism of UV-induced melanoma are 2-fold: DNA damage and oxidative stress. Accordingly, regulation of GADD45B may become a significant methodology to prevent melanoma formation. In the current study, we analyzed two sequencing datasets from one experiment. Differentially expressed genes (DEGs) and differentially expressed miRNAs (DEMs) were screened out. Both pathways and GO (Gene Ontology) enrichment analysis were performed and the DEG-DEM network was established. The relations between clinical data and specific factors were also analyzed based on TCGA database. The immune response in melanoma was also investigated in our study. Above all, we integrated genome-scale analyses of mRNA, miRNA, protein, mutation, methylation, and copy number (CN) via bioinformatics to identify new efficient biomarkers as potential therapeutic targets (Figure 1).

## Methods

### Sequencing Datasets

Dataset GSE80427, GSE80428, and GSE35387 were acquired from GEO Dataset. In this study, all experiments involving animals were approved by the Veterinary Office of the Canton Vaud (Switzerland). Mice were raised in a standard environment. Hairless female SKH-1 mice were 8–12 weeks old. Each experiment involved four animals per group assigned randomly and was repeated independently. Briefly, mice were exposed to a dose of 70 mJ/cm^2^ of UVB three times a week (24). Non-irradiated mice were used as controls. Then the skin was harvested and processed for mechanical epidermis/dermis separation (25).

Normal human epidermal melanocytes, HEMa-LP, were purchased from Life Technologies (Carlsbad, CA). The human malignant melanoma cell line A375 was purchased from American Type Culture Collection (Rockville, MD). A375 cells were maintained in Dulbecco's Modified Eagle Medium (DMEM), supplemented with 10% exosome-depleted fetal bovine serum (FBS) and penicillin (100 U/mL)/streptomycin (100 μg/mL). FBS was depleted of exosomes by ultracentrifugation at 100,000 × g for 16 h at 4°C. HEMa-LP cells were cultured in Medium 254 supplemented with Human Melanocyte Growth Supplement-2 (HMGS-2) in a 5% CO_2_ incubator at 37°C. All other cell culture reagents were obtained from Life Technologies. Exosomes were purified from culture supernatants by a three-step approach that includes ultrafiltration and ultracentrifugation (26).

### Microarray Data and Enrichment Analysis

Total RNA from tissues, cells and exosomes was isolated using Trizol extractions (Invitrogen). The RNA quantity was assessed by NanoDrop®ND-1000 spectrophotometer and RNA 6000 NanoChips with the Agilent 2100 Bioanalyzer (Agilent, Palo Alto, USA). On the one hand, 100 ng of total RNA was amplified using the Ambion® WT Expression Kit (4411973, Life Technologies). 5.5 μg of the cDNA was fragmented and labeled with the GeneChip® WT Terminal Labeling kit (901525, Affymetrix). On the other hand, small RNA libraries were prepared using 1 μg of total RNA according to the TruSeq Small RNA Sample Preparation Guide (Illumina, San Diego, CA). Libraries were sequenced either on Illumina HiSeq 2000 or HiSeq 2500 using v3 chemistry. To generate count data, the raw sequences were compared to mouse mature miRNA sequences (from miRBase version 17) and non-coding RNA sequences (Rfam version 10) by MEGABLAST.

Background deletion, quantile normalization, and probe assembly were performed. DEGs between NC (control group) vs. UVR (treatment group) samples were detected by the empirical Bayes method (27, 28) while DEMs were detected by the R package DESeq (29). *P*-values were adjusted for multiple comparisons using the Benjamini-Hochberg procedure (30). Both genes and miRNAs with adjusted *P* < 0.01 and logFC ≥ 1.0 were considered as differentially expressed. Gene and miRNA enrichment analyses were performed with DAVID version 6.7 (https://david.ncifcrf.gov/) and DIANA- mirPATH v.3 (http://diana.imis.athena-innovation.gr/DianaTools/index.php), respectively. The enriched biological GO and pathway terms were identified (31). The interaction network was drawn by Cytoscape. Some other databases used are listed in Table 1.

**Table 1:** List of database.

**Database ID**	**URL**
GEO dataset	https://www.ncbi.nlm.nih.gov/gds/?term=
TCGA	https://www.cancer.gov/
cBioportal of cancer genomics	https://www.cbioportal.org/
FireBrowe	http://firebrowse.org
DSA	http://cancer.digitalslidearchive.net/
The human protein atlas	https://www.proteinatlas.org/
Expasy	http://web.expasy.org/protparam/
PSIPRED	http://bioinf.cs.ucl.ac.uk/psipred/
Mexpress	https://mexpress.be/
Ensemble	http://asia.ensembl.org/index.html
Linked omics	http://www.linkedomics.org/
TransmiR v2.0	http://www.cuilab.cn/transmir
Targetscan	http://www.targetscan.org/vert_72/
OncomiR	http://www.oncomir.org/oncomir/index.html
TIMER	https://cistrome.shinyapps.io/timer/
STRING	https://string-db.org/
GEPIA	http://gepia.cancer-pku.cn/index.html
Pathview	https://pathview.uncc.edu/
TIP	http://biocc.hrbmu.edu.cn/TIP/index.jsp

### Statistical Analyses

Results are presented as mean values ± standard error of the mean (SEM). Unless mentioned otherwise, the statistical comparison between groups was performed by using *t*-test, a maximum of three comparisons were performed per panel, and robustness of statistical significance was verified after correction for multiple testing. Probability was considered to be significant at *p* < 0.05.

## Results

### DEGs and DEMs in UV Irradiated Mouse Skin

**Figure 2:**
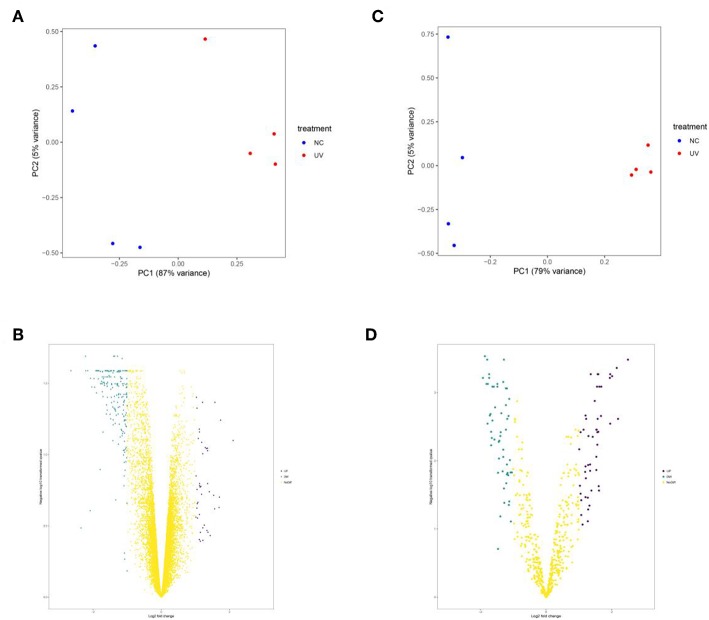
The principal component analysis **(A,C)** and volcano plots **(B,D)** of mRNA and miRNA datasets, respectively. In **(A,C)**, blue point represents sample in control group and the red one represents sample in UVR group. In **(B,D)**, the blue point represents down-regulated genes, the purple point represents up-regulated genes and the yellow one represents genes with insignificant change.

UVR damage in skin is mainly divided into two types, acute injury caused by high-dose UVR in a short time and chronic damage caused by perennial small-dose UVR. Generally, acute injuries can result in skin damage and sunburn while the chronic injury is the main cause of melanoma. Therefore, in our experiment, we selected mRNA and miRNA datasets in mouse sunburn model for analysis. After screening, 33 up-regulated genes, 185 down-regulated genes, 49 up-regulated miRNAs, and 55 down-regulated miRNAs were identified (Figure 2 and Figure S1). Then, pathway and GO enrichment analysis of DEGs and DEMs were performed in DAVID Database. There were 97 biological process (BP) terms, 25 cellular component (CC) terms, 32 molecular function (MF) terms, and 20 pathways associated with DEGs (Figure S2). Meanwhile, the DEMs were involved in 24 BP terms, 9 CC terms, 5 MF terms, and 84 pathways (Figure S3). Notably, 29 increased miRNAs and 28 reduced miRNAs were enriched in melanoma pathway (Figures S4, S5). The results further confirmed that UV damage is associated with melanoma formation.

### MiR-300 Targets Gadd45b in Melanoma Pathway

**Figure 3:**
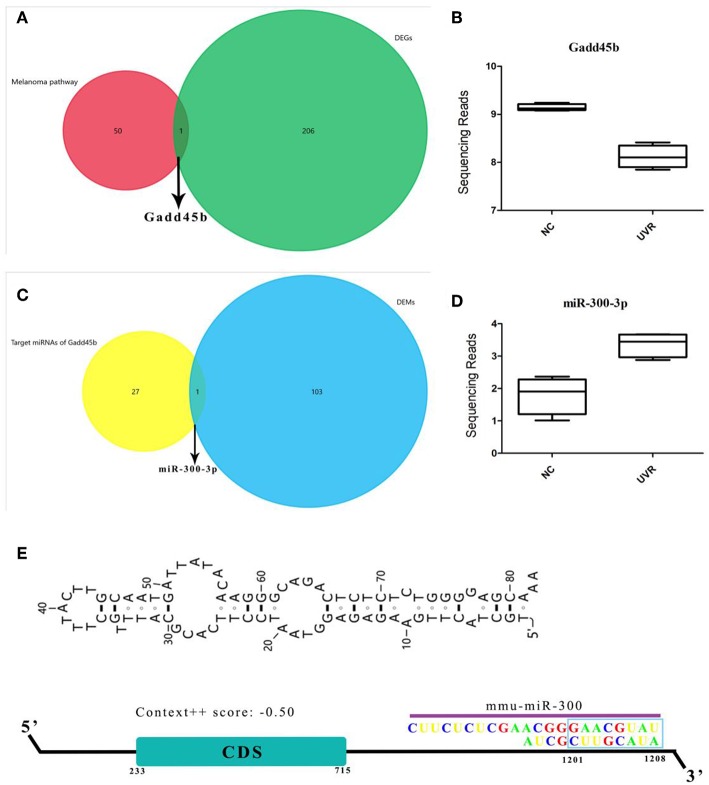
Gadd45b is a target gene of miR-300. **(A–D)** Shows the result of a comparison between different datasets and the expression of Gadd45b and miR-300. **(E)** Sketch map of miR-300 binding site in Gadd45b 3′-UTR.

Melanoma pathway plays a key role in melanocyte cancer pathogenesis. To further study the effects of UVR on the melanoma pathway, we analyzed the expression profiles of 73 genes involved in the melanoma pathway in a mouse model. Only the Gadd45b was significantly down-regulated (Figure 3 and Figure S6). Interestingly, miR-300, target to 3′-UTR of Gadd45b, was significantly increased after UVR (Figure 3E). In addition, more genes in the melanoma pathway were targeted by DEMs, and their expression remains the same without obvious changes (Figure S7). However, miR-300 is not enriched in the melanoma pathway, suggesting that its main function is not to regulate melanoma pathway. Only after exposure to UV abnormal expression and regulation of Gadd45b appeared, indicating that miR-300 just responds to oxidative stress or DNA damage of UVR.

### Gadd45b in Melanoma

After analysis of DEGs and DEMs, it was supposed that miR-300 and GADD45B might regulate melanoma development. However, studies about GADD45B and melanoma are very infrequent, and the mechanism is still unclear. To fully study the function of GADD45B, we comprehensively analyzed its expression profile and protein feature. GADD45B has four isoforms. The subtype recorded without defined 3D structure. Thus, the information of GADD45B contains secondary structure and characters were predicted (Figure S8). The results showed that the GADD45B is unstable and the half-life is 30 h *in vitro*. Immunohistochemical results of skin tissue showed that GADD45B was expressed in various skin cells, especially in the epidermis. The expression of GADD45B in melanoma was also showed (Figures S9B,C). In addition, by immunofluorescence assay in different cell lines, it was found that GADD45B is localized in nucleus (Figure S9A). However, whether GADD45Bs take part in cancer development is unclear. Therefore, the expression profiles of GADD45B in different tissues and different tumors were compared (Figures S10A,B). There was no gender difference in the expression of GADD45B. Moreover, GADD45B had lower level in melanoma compared with normal melanocytes (Figure S10C). It was hypothesized that, once melanoma formed, caused by cancer's self-defense, GADD45B would be down-regulated because of its tumor suppression.

**Figure 4:**
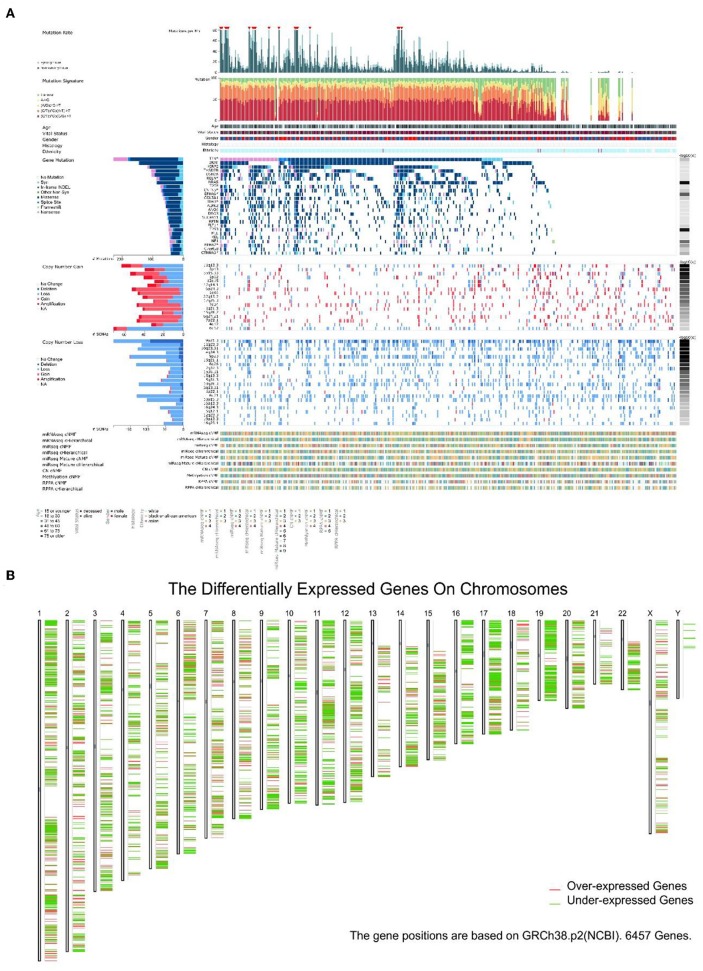
The landscape of Genomic Alterations in melanoma. **(A)** Integrated plot of clinical and molecular features for all melanoma samples. From top to bottom panels indicate: frequency of mutations per Mb (megabase); mutational signatures, indicating type of substitution; clinical data; gene mutation; copy number alterations including gains (pink), amplification (red), shallow deletion (pale blue), or deep deletion (dark blue); mRNAseq consensus non-negative matrix factorization (cNMF); mRNAseq cHierarchical; miRseq cNMF; miRseq cHierarchical; miRseq mature cNMF; miRseq mature cHierarchical; CN cNMF; methylation cNMF; Reverse phase protein arrays (RPPA) cNMF; and RPPA cHierarchical. The key to the color-coding is at the bottom. **(B)** 6,457 genes expression in 24 chromosomes based on GRCh38.p2, red lines represent over-expressed genes and green lines represent under-expressed genes.

It is known that the reason UV could become the primary factor of melanoma is DNA damage. Gene mutations in melanoma were studied in 368 patients (87 patients without mutation) (Figure 4A). The top 25 mutated genes like BRAF (96.58% missense), TP53 (58.33% missense), and XIRP2 (77.12% missense) were listed. In addition, mutation rate, mutation signature, clinical data, copy number (CN), and other related data were also shown. In melanoma, gene mutations were relatively rare while gene dysregulation was common in 24 chromosomes (Figure 4B).

**Figure 5:**
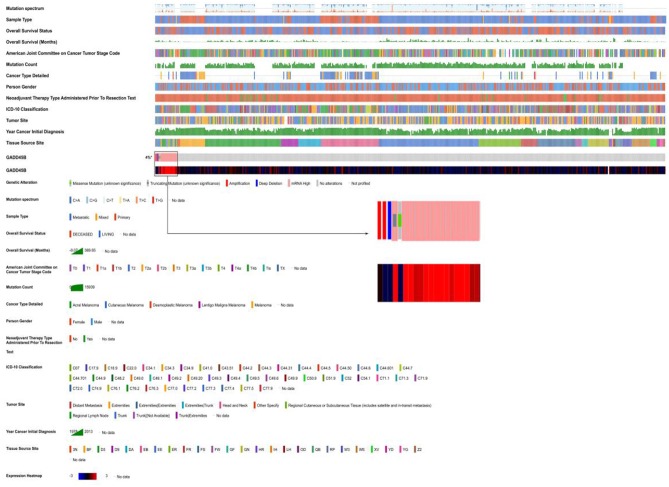
GADD45B in melanoma patients based on TCGA. Integrated plot of clinical data and GADD45B mutation in 472 melanoma samples. From top to bottom panels indicate: overall survival, American Joint Committee on Cancer tumor stage code, mutation count, cancer type detailed, person gender, neo-adjuvant therapy type administered prior to resection text, ICD-10 classification, tumor site, year cancer initial diagnosis, tissue source site, mutation symbol of GADD45B, heatmap of GADD45B. The key to the color-coding is at the bottom.

In order to study the relation between GADD45B mutation and melanoma, multi-omics data of 473 patients in TCGA database were analyzed (Figure 5). As a result, 4% patients were identified with GADD45B mutation including 2 CN amplification, 1CN deep deletion, 1 missense mutation, 1 truncated mutation, and 15 with high mRNA level. Histopathologic sections of each sample with GADD45B alteration were showed in Figure S11. Methylation is often associated with gene expression and cancer development. According to the methylation analysis of GADD45B, it suggested GADD45B methylation had a correlation with GADD45B expression (*R*^2^ = 0.04) and overall survival (*R*^2^ = 0.01) (Figures S12, S13). Moreover, a total of 11 types methylations were identified and once cg16624646 appeared, melanoma patients will have worse prognosis (Figure S14 and Table 2). The other correlations were also calculated like GADD45B mutation vs. overall survival. It demonstrated that the status of GADD45B might be involved in melanoma development. As mentioned above, in melanoma, GADD45B could interact with other genes like STAT3. To further verify the role of GADD45B in melanoma, the correlation between GADD45B and these co-expression genes was calculated (Figure S15). GADD45B had negative correlation with Nrf2 (*R*^2^ = 0.04), in contrast with Jiang's study (22). This was probably caused by the altered regulation pattern of GADD45B-network in cancer environment (Figure S16). GADD45B is a key factor in melanoma, and once alteration appeared, the prognosis of patients becomes worse. The median survival of cases with alteration is 48.82 months while cases without alteration are 79.53 months (*p* = 0.515). In addition, the median disease-free of cases with alteration was 24.8 months while cases without alteration were 51.48 months (*p* = 0.0733) (Figure S17).

**Table 2:** Information of 11 types GADD45B methylation in skin melanoma.

**Name**	**Hazard ratio (HR)**	**Confidence interval (CI)**	***P*-value**	**LR test_pvalue**	**PH test_Pvalue**	**Best_split**	**MAPINFO**	**UCSC RefGene_Group**	**Relation_to_UCSC CpG_Island**
cg00014806	0.921	(0.708; 1.2)	0.54	0.54	0.55	Median	2475658	TSS1500	Island
cg01485266	1.225	(0.904; 1.661)	0.19	0.18	0.86	q25	2476132	TSS200	Island
cg08259617	1.239	(0.89; 1.725)	0.2	0.19	0.16	q25	2476129	TSS200	Island
cg08682582	1.226	(0.941; 1.596)	0.13	0.13	0.074	Mean	2476077	TSS200	Island
cg13666550	1.226	(0.939; 1.602)	0.14	0.14	0.36	Mean	2475637	TSS1500	Island
cg14733855	1.105	(0.847; 1.442)	0.46	0.46	0.14	Median	2477194	Body	Island
cg16624646	1.302	(0.999; 1.698)	0.051	0.051	0.8	Mean	2474993	TSS1500	Island
cg19257531	0.783	(0.583; 1.051)	0.1	0.11	0.22	q25	2475640	TSS1500	Island
cg19351423	1.097	(0.805; 1.495)	0.56	0.55	0.83	q25	2476226	5′UTR;1stExon	Island
cg22246692	0.844	(0.62; 1.148)	0.28	0.29	0.95	q25	2475189	TSS1500	Island
cg22376929	0.842	(0.619; 1.146)	0.28	0.28	0.54	q25	2478127	3′UTR	N_Shore

### Melanocytes Transport miR-300 Through Exosomes

**Figure 6:**
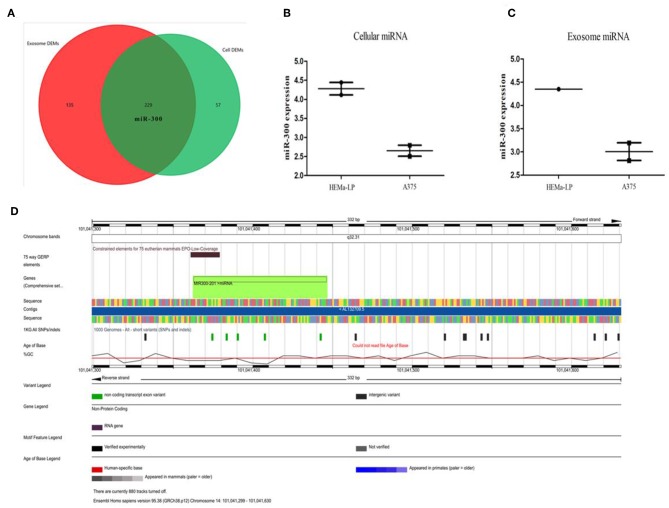
TFs and miR-300 in melanoma. **(A–C)** Venn diagram comparing melanocytes vs. melanoma and melanocyte exosomes vs. melanoma exosomes. The red circle represents the DEMs in exosome and the green circle represent the DEMs in cells. Sequencing data was showed in the box-plot. **(D)** Integrated plot of miR-300. The key to the color-coding is at the bottom.

It has been predicted that miR-300 could bind to GADD45B 3′-UTR and the expression profiles in UV irradiated skin were in line with the forecast. However, in keratinocytes, it was doubted that miR-300 might be partly derived from surrounding cells. As is it known, in the epidermis, melanocytes could communicate with other cells (e.g., keratinocytes) via exosomes. In GSE35387 dataset, miR-300 was identified as down-regulated in melanoma and exosomes when compared with normal melanocytes (Figure 6 and Figure S18). Therefore, it was supposed that in UV irradiated keratinocytes of melanoma patients, Gadd45b would be up-regulated, given the lower level miR-300. The location of miR-300 is in chromosome 14-NC_000014.9 (101041363-101041445) and acts as a tumor suppressor in several cancers. Nevertheless, in melanoma patients, miR-300 could result in worse prognosis (Figure S19). In order to find out the reasons behind miR-300 regulation, transcription factors (TFs) bind to miR-300 promoter were predicted (Figure S20A). Among these TFs, TP53, JUN, JUNB, FOS, FOSB not only regulated miR-300, but also interacted with GADD45B (Figure S20B). Moreover, MYC (log FC = −3.32), PPARG (log FC = 2.17), ZIC2 (log FC = 3.62) were also dysregulated in melanoma (Figure S20C). It is well-known that MYC is an oncogene and TP53 plays an important role in various cancers. Therefore, these eight specific TFs might be involved in the regulation of melanoma along with miR-300. In 472 melanoma patients, 21% have TP53 alteration, 5% have FOS alteration, 5% have FOSB alteration, 6% have JUN alteration, and 5% have JUNB alteration. Moreover, 15% have MYC alteration, 8% have PPARG alteration, and 2.6% have ZIC2 alteration (Figure S21). The overall survival of TFs was also calculated (Figure S22). The results showed that the specific TFs might interact with miR-300/GADD45B in melanoma progression.

**Figure 7:**
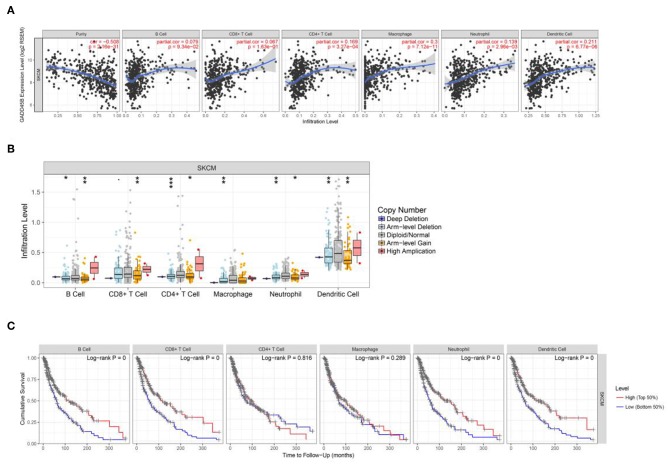
The interaction of immune cell infiltration and specific factors. **(A,B)** The correlation of expression level vs. immune infiltration and copy number vs. immune infiltration using two-sided Wilcoxon rank sum test. The analysis contain B cell, CD8+ T cell, CD4+ T cell, macrophage, neutrophil and dendritic cell. **(C)** Visual survival differences of various immune cells (B cell, CD8+ T cell, CD4+ T cell, macrophage, neutrophil, and dendritic cell) in melanoma. The split percentage of patients is from 5 to 50%; the survival time interval is from 0 to 200 months. The red line represents high level and the blue line represent low level.

Immune infiltration is an important indicator of cancer development. In the present study, through bioinformatics analysis, some specific factors have been identified. After the prediction in TIMER database, it is suggested that the immune infiltrates of B cell, CD8^+^ cell, CD4^+^ cell, macrophage, neutrophil, and dendritic cell correlate with melanoma patient survival. In addition, the gene feature (expression level and CN) of each factor were predicted to regulate immune infiltration (Figure 7 and Figure S23). Cases with high or low immune infiltration scores typically showed coordinate increases or decreases in multiple inflammatory cells, rather than in single cell type. We also analyzed tumor immunophenotype of patients with gene alteration like GADD45B (Figure S24). Taken together, the data suggest that these factors might regulate immune response and act as immune microenvironment biomarkers in melanoma.

## Discussion

Melanoma is a common malignant skin tumor with many different causes. As well-known, the oxidative stress and DNA damage caused by UV are the major causative factors of melanoma, while the involved molecular mechanism is still unclear (32–35). This study attempts to explain the molecular mechanism and screen new biomarkers.

Firstly, in the UV chronic injury mouse model, DEGs and DEMs were screened by analyzing two sequencing datasets followed by pathway and GO enrichment analysis. GADD45B inhibits cancer development in a variety of situations (36). There are also studies that have confirmed that GADD45B could effectively respond to oxidative stress (22). In addition, GADD45B is certainly involved in DNA damage as a member of the GADD45 family. Thus, we believe GADD45B may be a key factor in the regulation of melanoma against oxidative stress and DNA damage. After analyzing the relation between GADD45B and melanoma according to TCGA database, it was also confirmed that gene features of GADD45B might correlate with poor prognosis.

MiR-300 may also play a significant role in melanoma via inhibiting GADD45B expression. Interestingly, after analyzing sequencing data, we found that melanocytes could export miR-300 via exosomes. In addition, miR-300 was significantly down-regulated in melanoma cells and exosomes compared with normal melanocytes. Although ROSs produced by UV is the main cause of melanoma initiation, they kill melanoma cells. In summary, we suspect that miR-300 would be increased in melanoma cells and exosomes, thereby upregulating GADD45B in keratinocytes and melanoma cells, protecting melanoma against ROS. However, miR-300 may not play a single role in melanoma due to the particularity of miRNA regulation. Recently, studies confirmed that KDM5A and KDM6A can act as oxygen molecule receptors to mediate H3K4me3 demethylation (37, 38). However, as a member of the same family, KDM5B plays the opposite role. It indicated that KDM5B might promote H3K4me3 methylation. Importantly, KDM5B is a predicted target gene of miR-300. In addition, histone methylation could lead to chromatin remodeling, which in turn inhibits cell proliferation. Therefore, it is assumed that miR-300 might promote cell proliferation via KDM5B.

**Figure 8:**
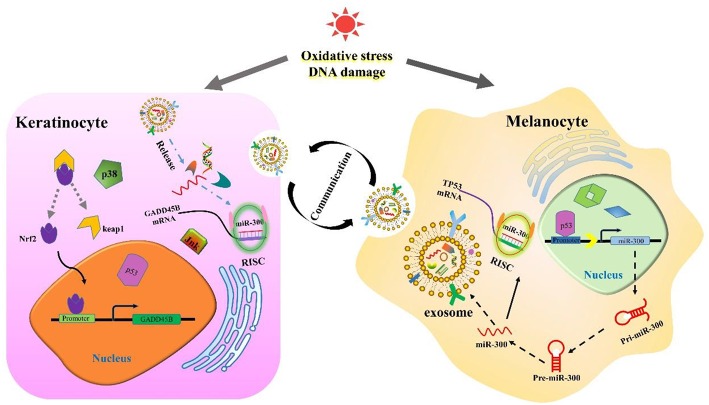
The cartoon diagram of communication between keratinocyte and melanocyte.

Previous studies have demonstrated that when external stimuli cause DNA damage, TP53 will respond first. TP53 can inhibit the expression of GADD45B, but the promoting response element has not been confirmed (39). It was also demonstrated that TP53 regulated various pathways and molecules in melanoma pathogenesis (40). According to the analysis, miR-300 might mediate TP53 regulation of GADD45B (Figure 8). Interestingly, it has been confirmed that TP53 can bind to miR-300 promoter and regulate its expression. Meanwhile, TP53 is a predicted target of miR-300 (41, 42). The TFs that regulate miR-300 and GADD45B also correlate with immune cell infiltration, which is one of the main indicators and physiological responses to melanoma. This study provides a detailed TFs-mRNA-miRNA axis in melanoma available for further research. The results improve our understanding of melanoma formation and could hopefully lead to new therapeutic approaches for this deadly disease.

## Data Availability Statement

Publicly available datasets were analyzed in this study. This data can be found here: https://www.cancer.gov/about-nci/organization/ccg/research/structural-genomics/tcga.

## Ethics Statement

All experiments were approved by the local ethics committee. These sequencing datasets were obtained from GEO database, a public database.

## Author Contributions

LC and LZ contributed to the conception and design of the work. LC analyzed data and wrote this manuscript. LZ contributed to interpretation of data for the work. HL and VK contributed to the revision of manuscript for important intellectual content. All authors contributed to final approval of the version to be published and agree to be accountable for all aspects of the work.

### Conflict of Interest

The authors declare that the research was conducted in the absence of any commercial or financial relationships that could be construed as a potential conflict of interest.
