# Supplementary material for: MicroRNA-300: A Transcellular Mediator in Exosome Regulates Melanoma Progression
Source: Front Oncol. 2019 Oct 15;9:1005. doi: 10.3389/fonc.2019.01005 (PMC6803498; doi:10.3389/fonc.2019.01005)
Supplement: Supplementary file 1 [file Data_Sheet_1.docx]

**Additional supplementary material of “MicroRNA-300：A transcellular mediator in exosome regulate melanoma progression”**


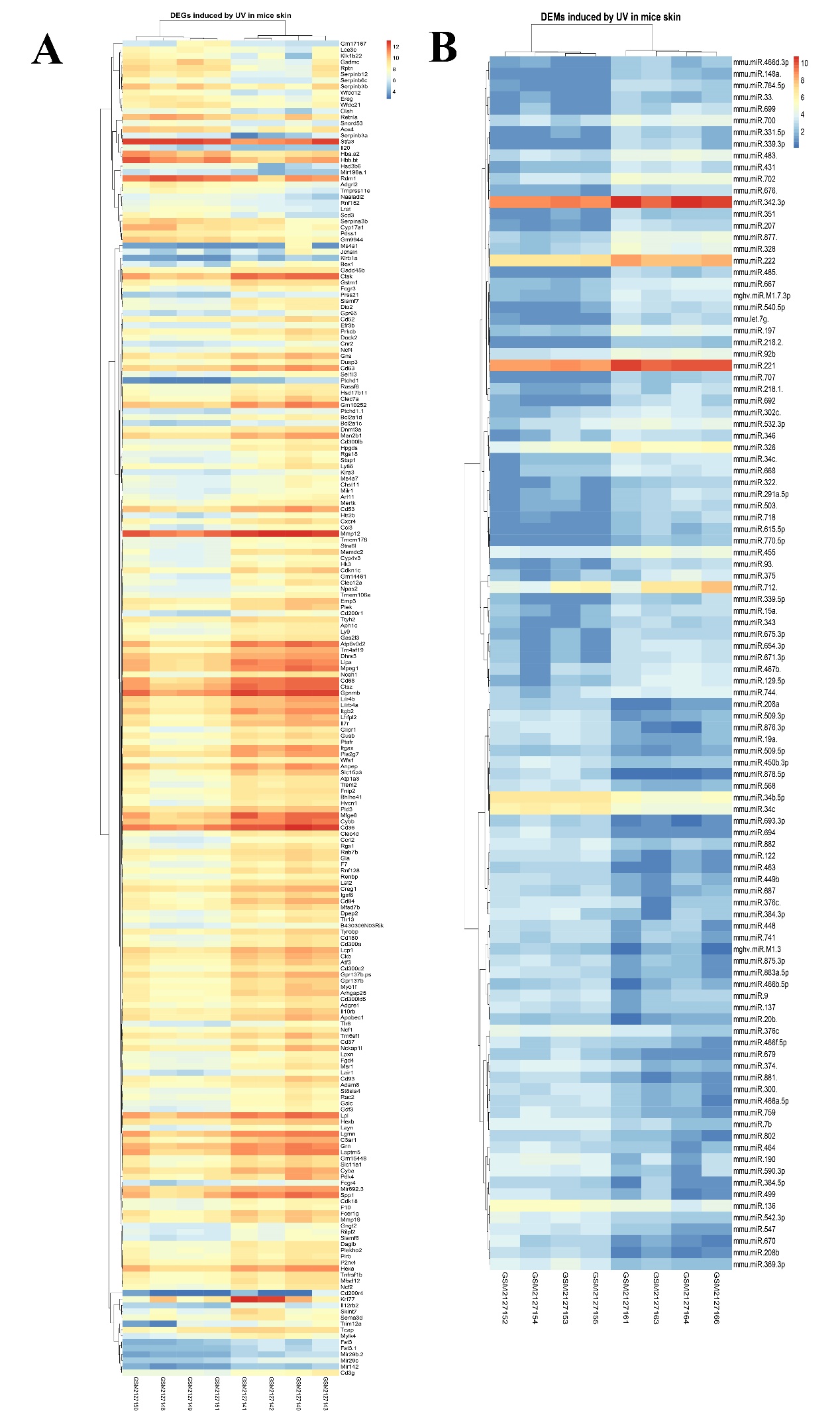


**Figure S1. The heat maps show DEGs (A) and DEMs (B) in the UVR group.** Color depth represents expression.


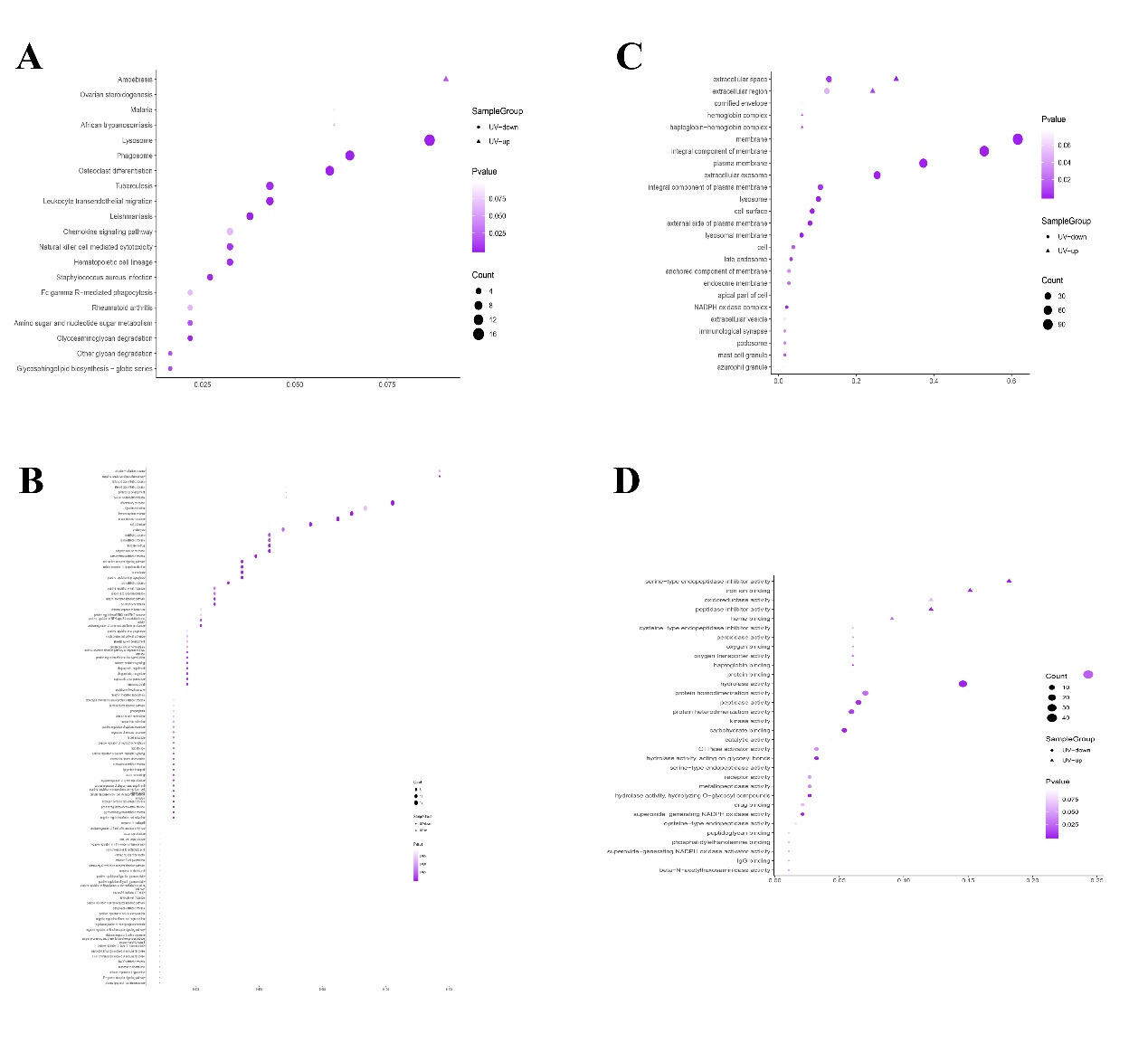


**Figure S2. The enriched GO terms and pathways of DEGs. A,** The enriched pathways of DEGs. **B-D,** The enriched BP, CC and MF items of DEGs were shown respectively. Color depth represents p-value and the diameter represent the number of enriched genes. The circle represents the down-regulated genes in UVR group and the triangle represents up-regulated genes in UVR group.


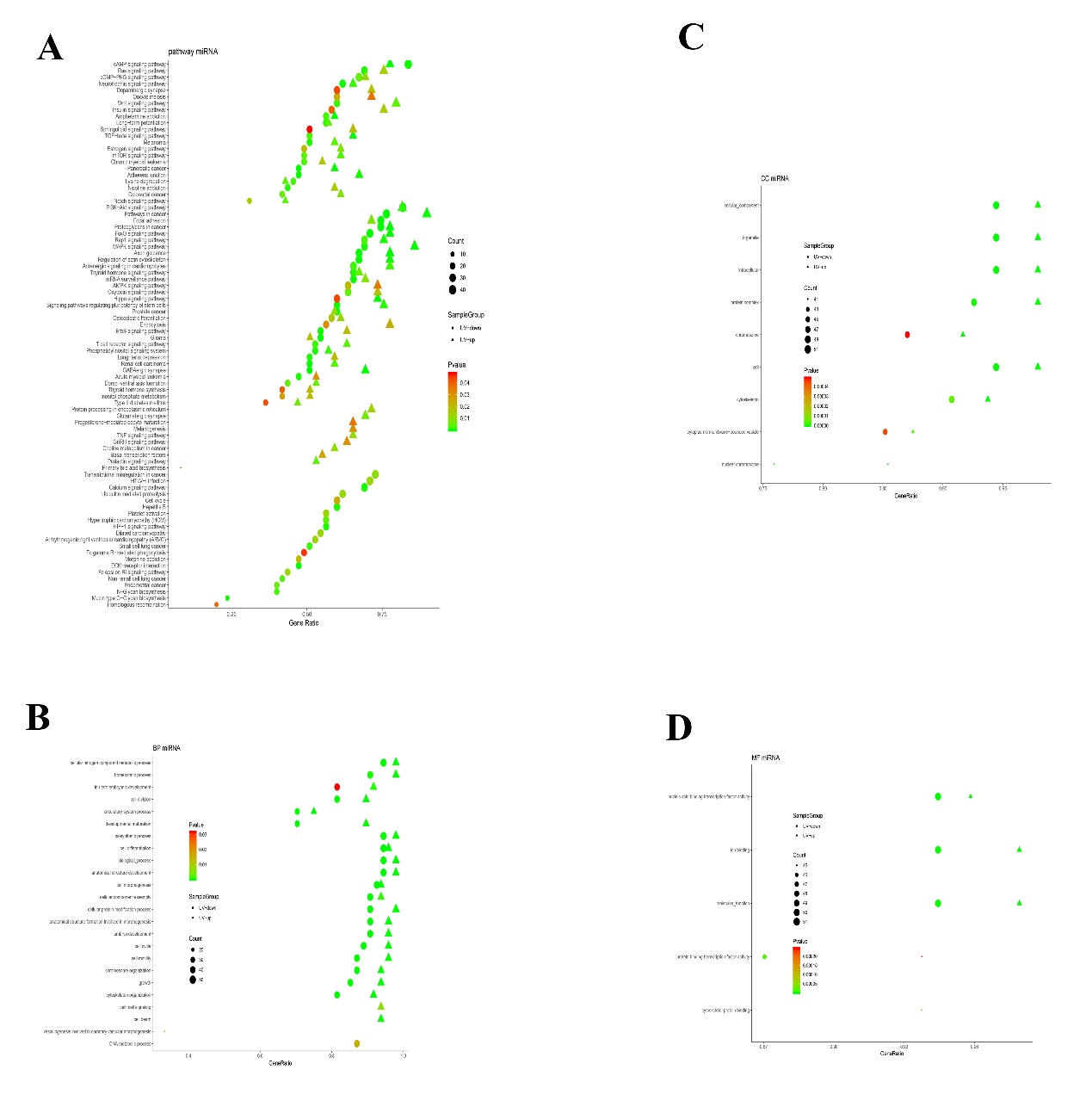


**Figure S3. The enriched GO terms and pathways of DEMs. A,** The enriched pathways of DEMs. **B-D**, The enriched BP, CC and MF items of DEMs were shown respectively. Color depth represents p-value and the diameter represent the number of enriched miRNAs. The circle represents the down-regulated miRNAs in UVR group and the triangle represents up-regulated miRNAs in UVR group.


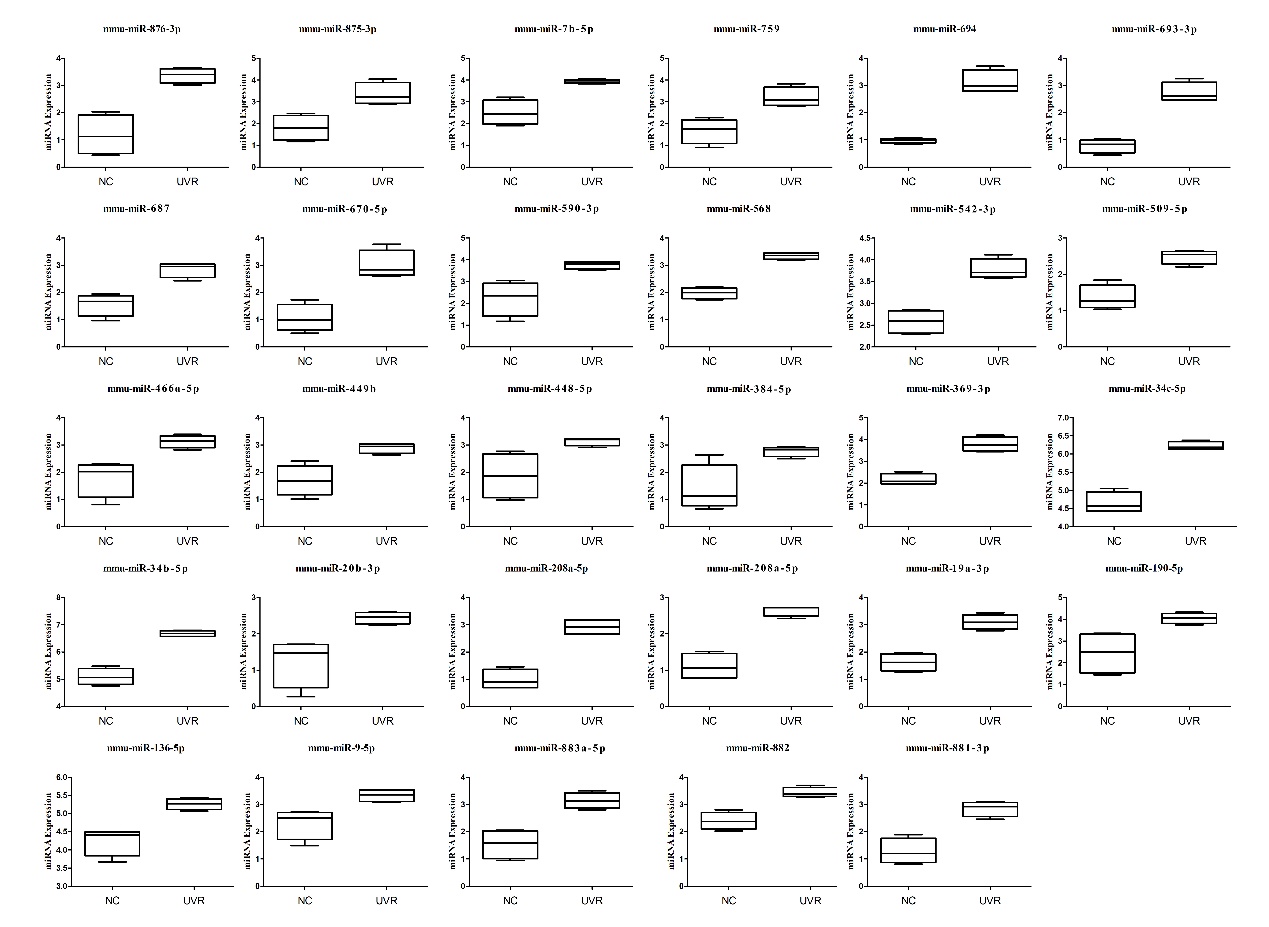


**Figure S4. The expression of up-regulated miRNAs enriched in melanoma.**


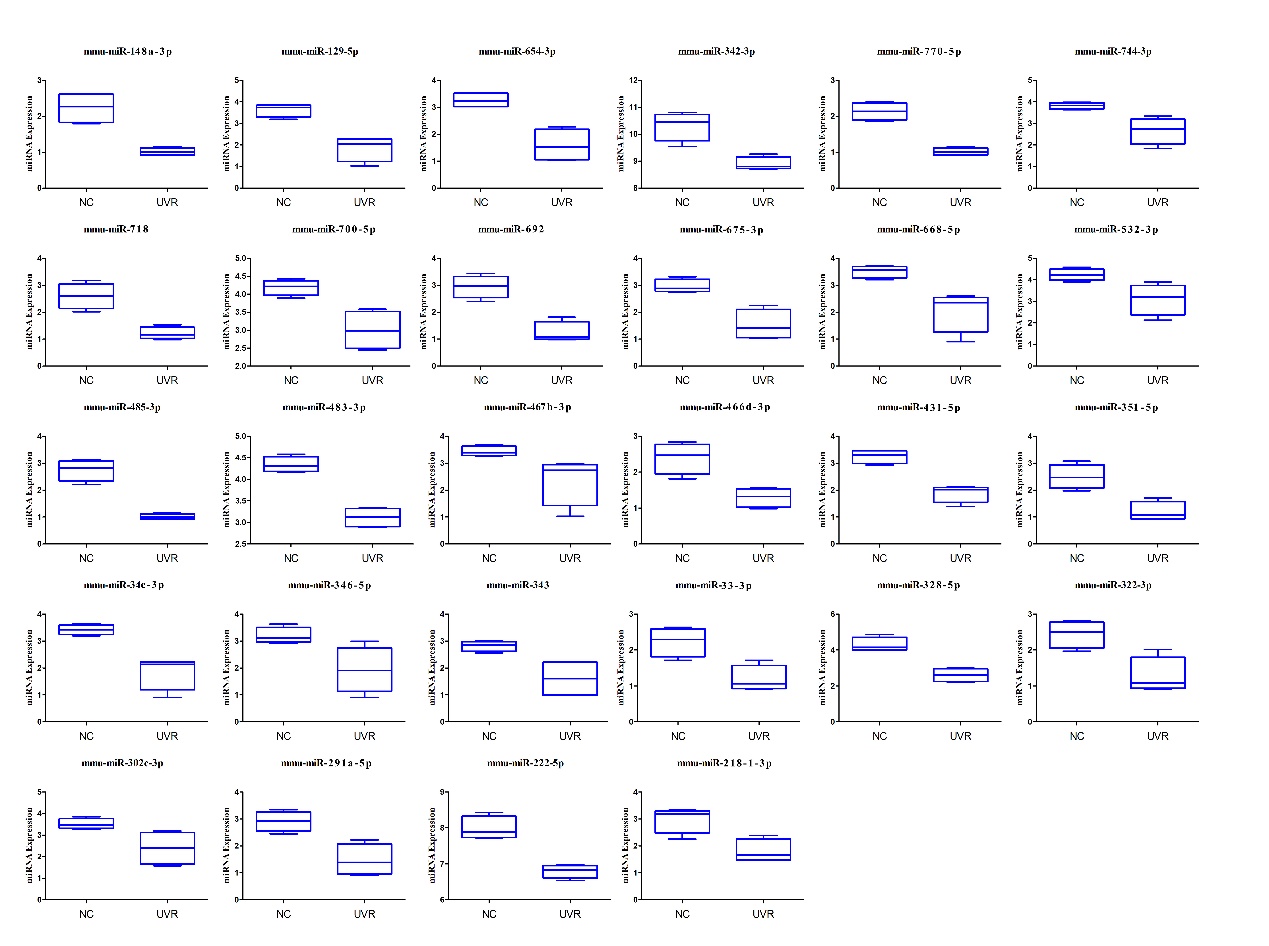


**Figure S5. The expression of down-regulated miRNAs enriched in melanoma.**


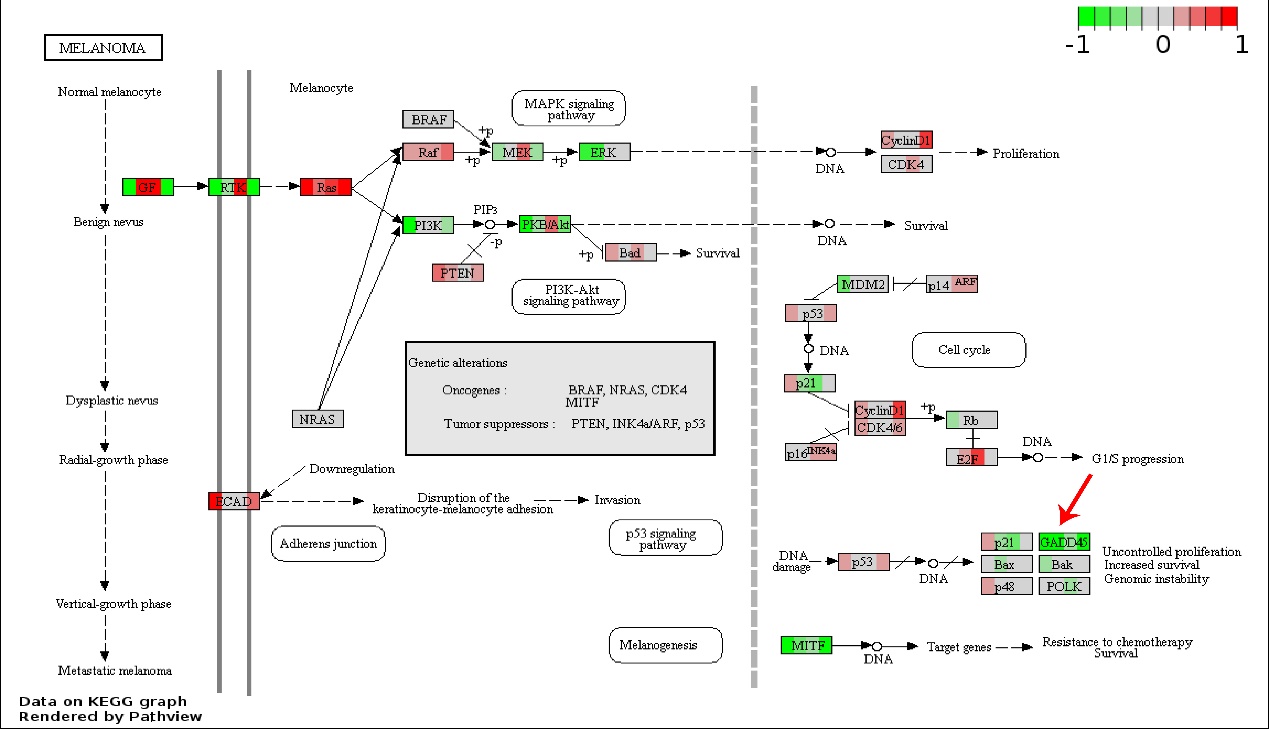


**Figure S6. Sketch map of melanoma pathway.** Red indicates upregulated gene and green indicates downregulated gene.


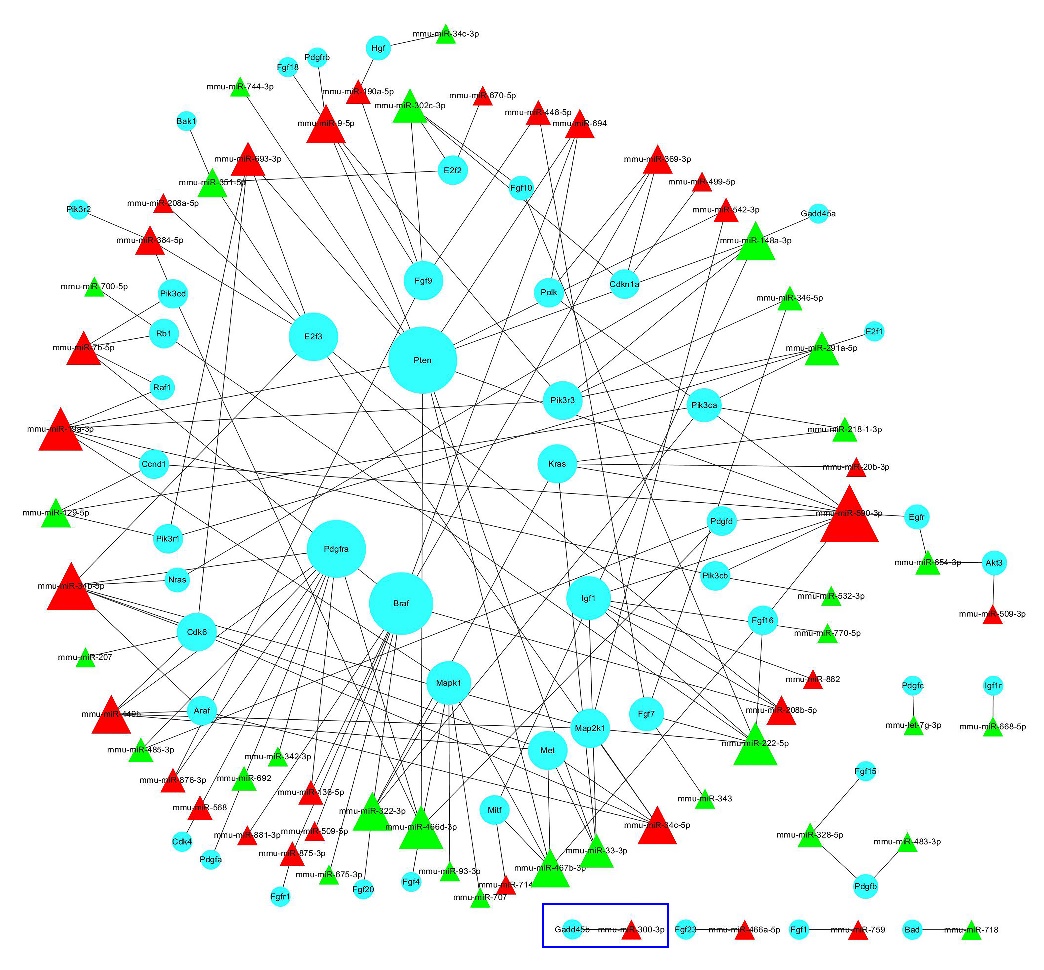


**Figure S7. The network of genes in melanoma and DEMs.** Red triangles represent upregulated miRNAs and green triangles represent downregulated ones. Blue circles with different diameter represent genes in melanoma pathway with the different number of target miRNAs.


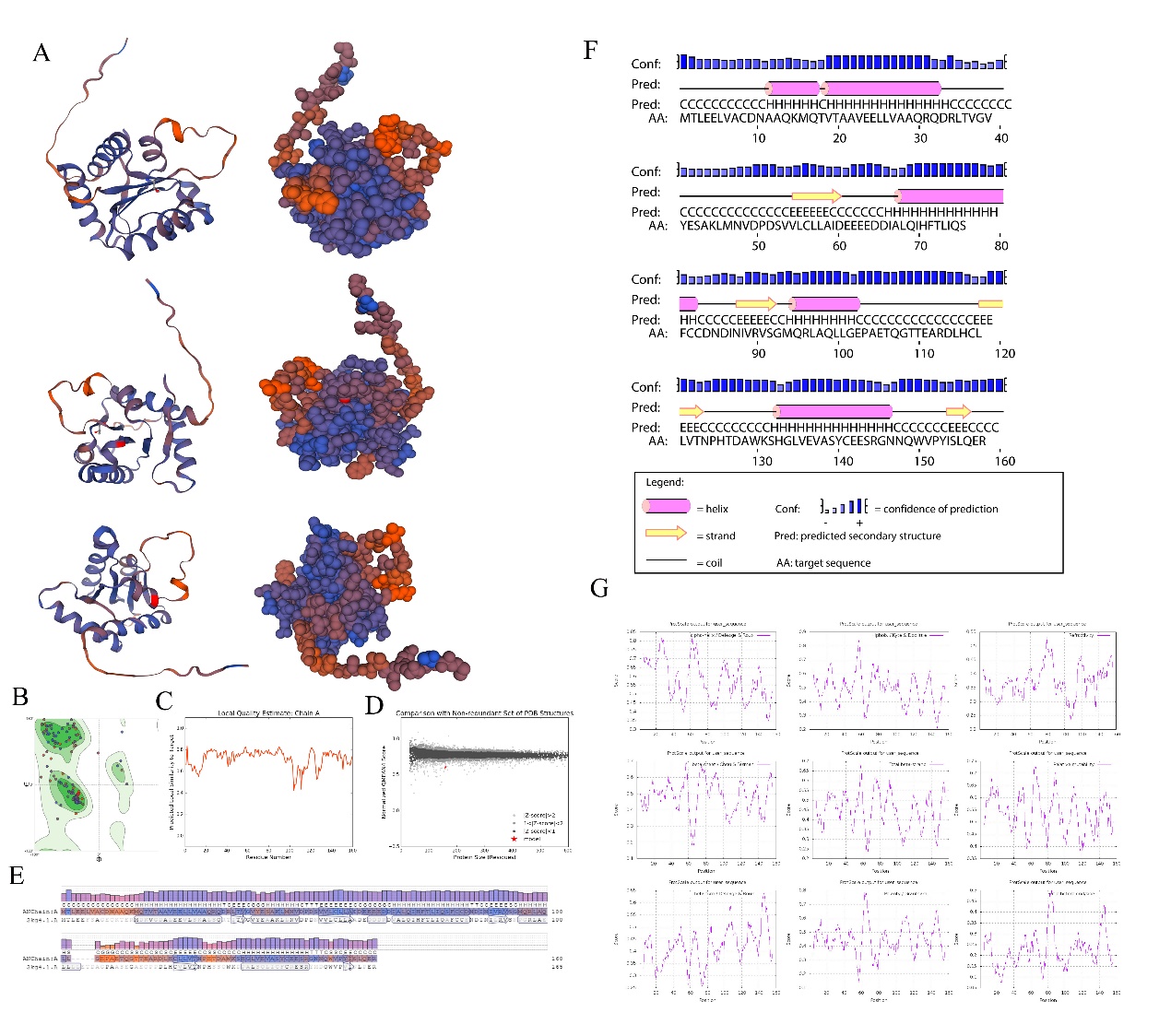


**Figure S8. Structure prediction of GADD45B. A,** Predicted 3D structure of GADD45B at different angles via homology modeling based on GADD45A. **B-D** show ramachandran Plots, local quality estimate and PDB structures comparison of 3D structure prediction. **E,** sequence alignment between GADD45B and GADD45A. **F,** shows secondary structure of GADD45B. **G,** Analysis of α-helix, β-sheet, β-turn, relative mutability of amino acid, ratio of hetero end/side, polarity, refractivity, total β-strand and hydrophilia in GADD45B sequence.


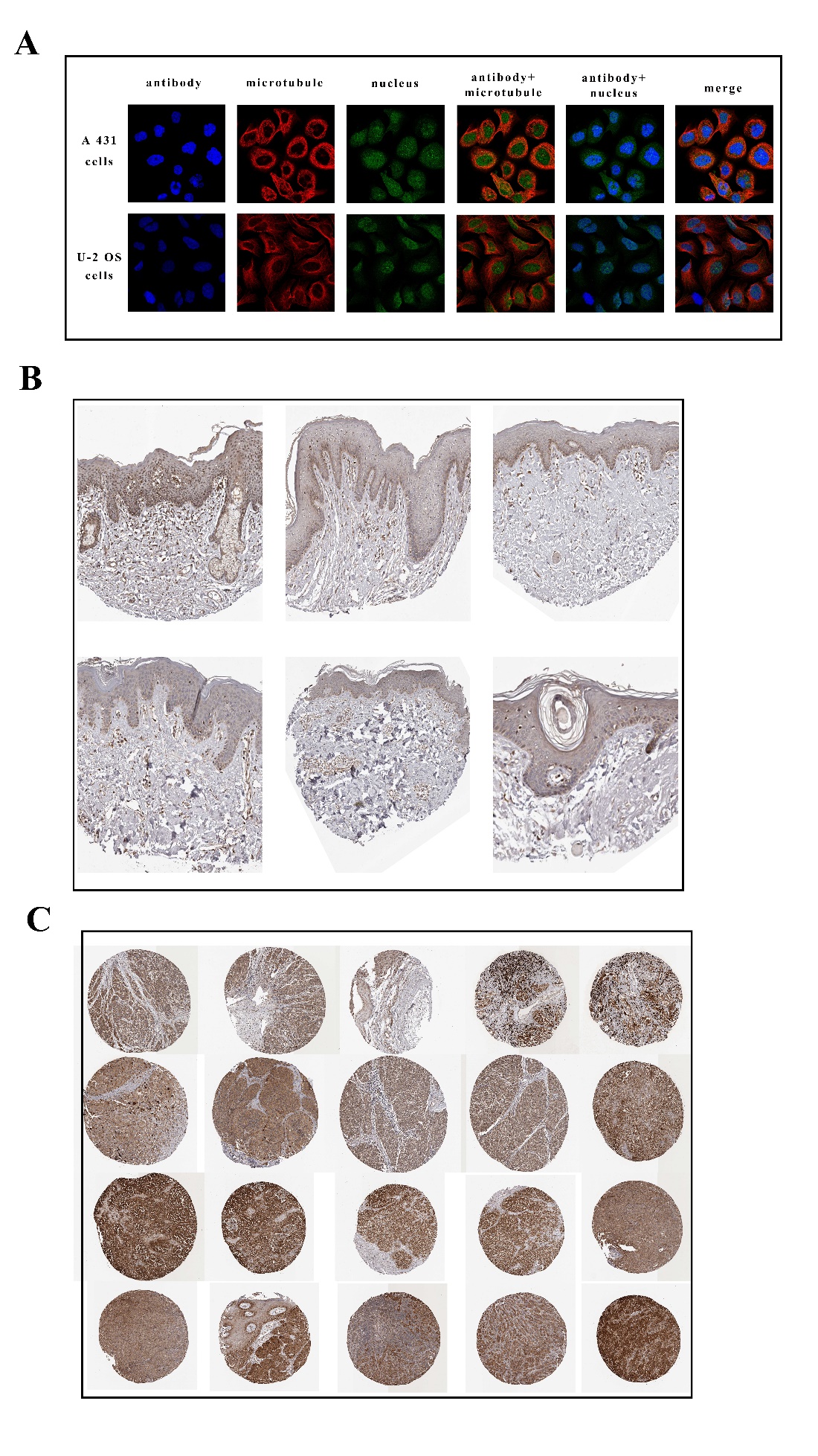


**Figure S9. The** **location of GADD45B in cells and tissue. A,** Immunofluorescence pictures of GADD45B in A431 (Epidermoid carcinoma) and U-2 os (Human osteosarcoma) cell lines**. B,** Immunohistochemical pictures of GADD45B in skin tissue**. C,** Immunohistochemical pictures of GADD45B in melanoma**.**


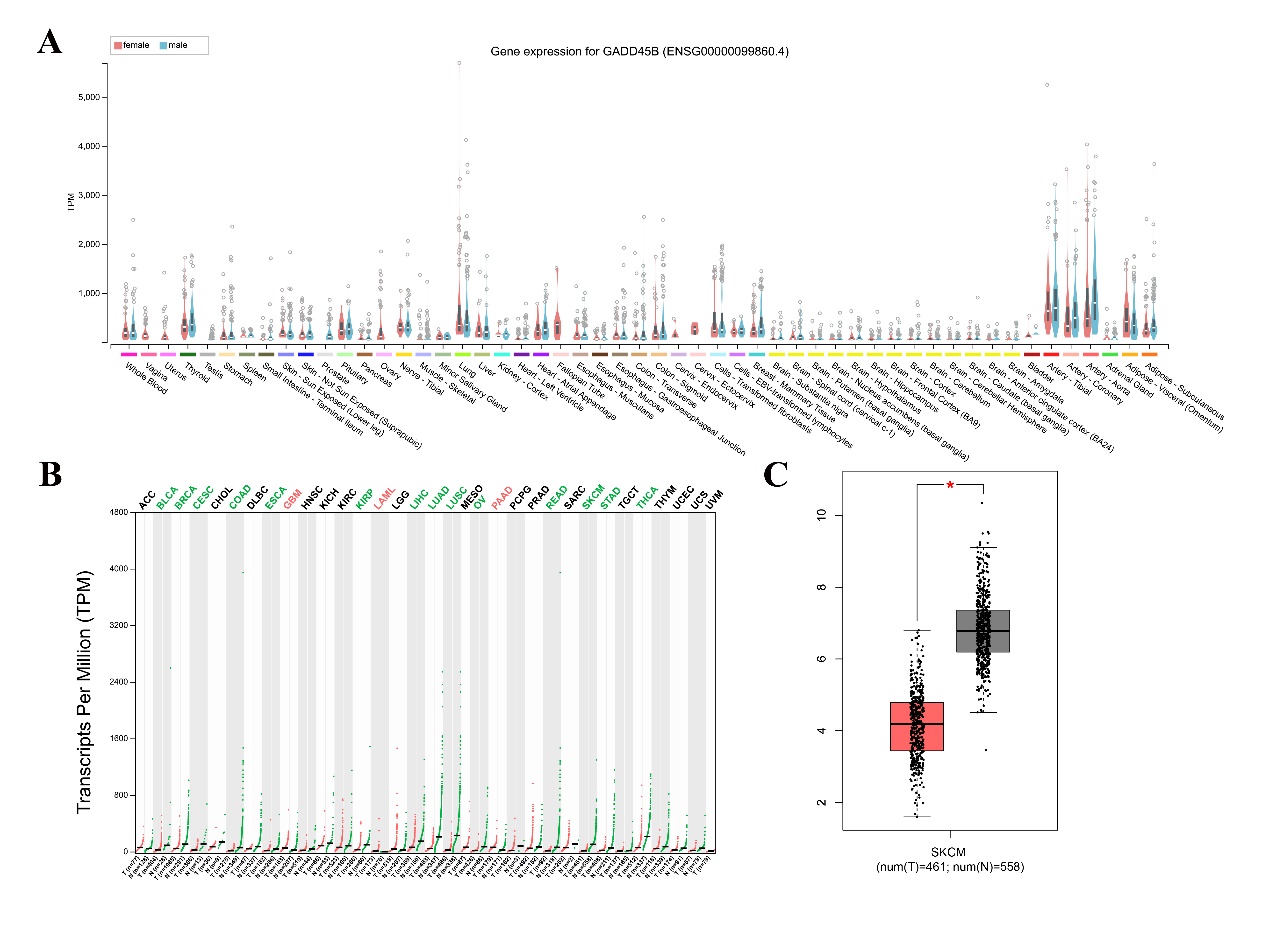


**Figure S10. The expression profiles of GADD45B in different organs. A,** Transcripts per million (TPM) of GADD45B in different organs. Red represent female and blue represents male. Each gray circle represent a single sample. In X-axis, different colors represent different organs. **B,** Green dots represent normal cells and red dots represent tumor cells. **C,** Box plot of melanocyte (558 samples) vs. melanoma (461 samples).


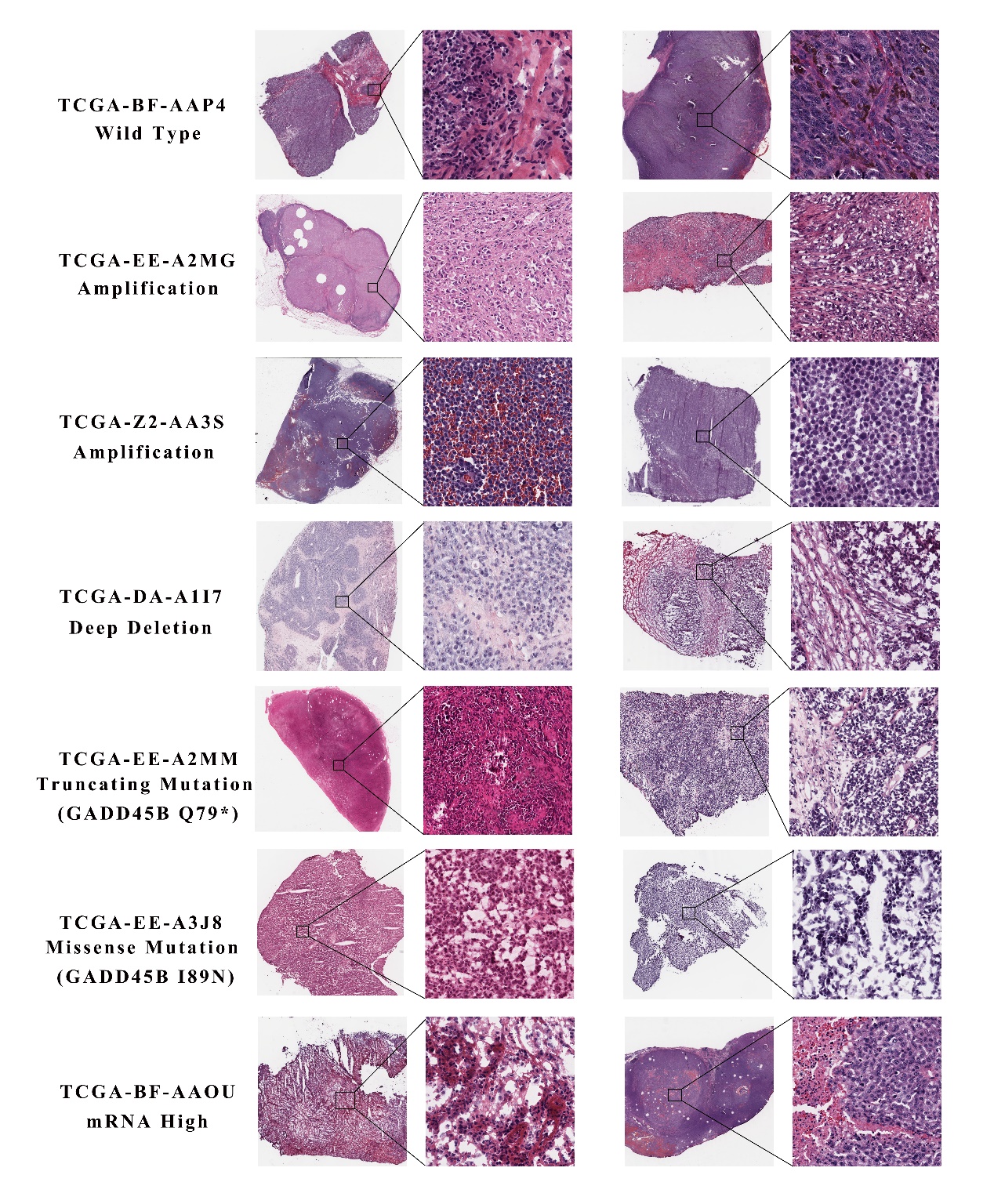
 **Figure S11. Histopathologic sections of each sample with GADD45B alteration.** From top to bottom are TCGA-BF-AAP4 (wild type), TCGA-EE-A2MG (amplification), TCGA-Z2-AA3S (amplification), TCGA-DA-A1I7 (deep deletion), TCGA-EE-A2MM (truncating mutation-Q79*)， TCGA-EE-A3J8 (missense mutation-I89N) and TCGA-BF-AAOU (mRNA high).


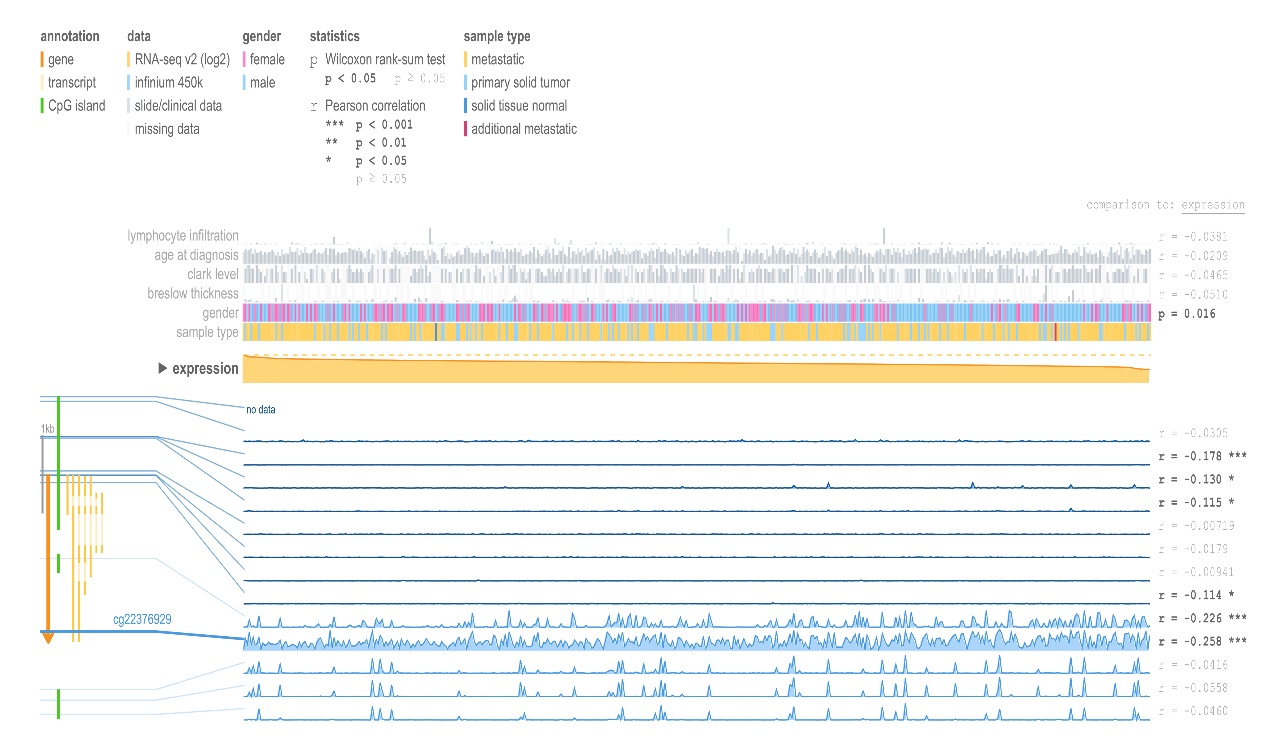


**Figure S12. Methylation analysis of GADD45B.** The key to the color-coding is at the top.


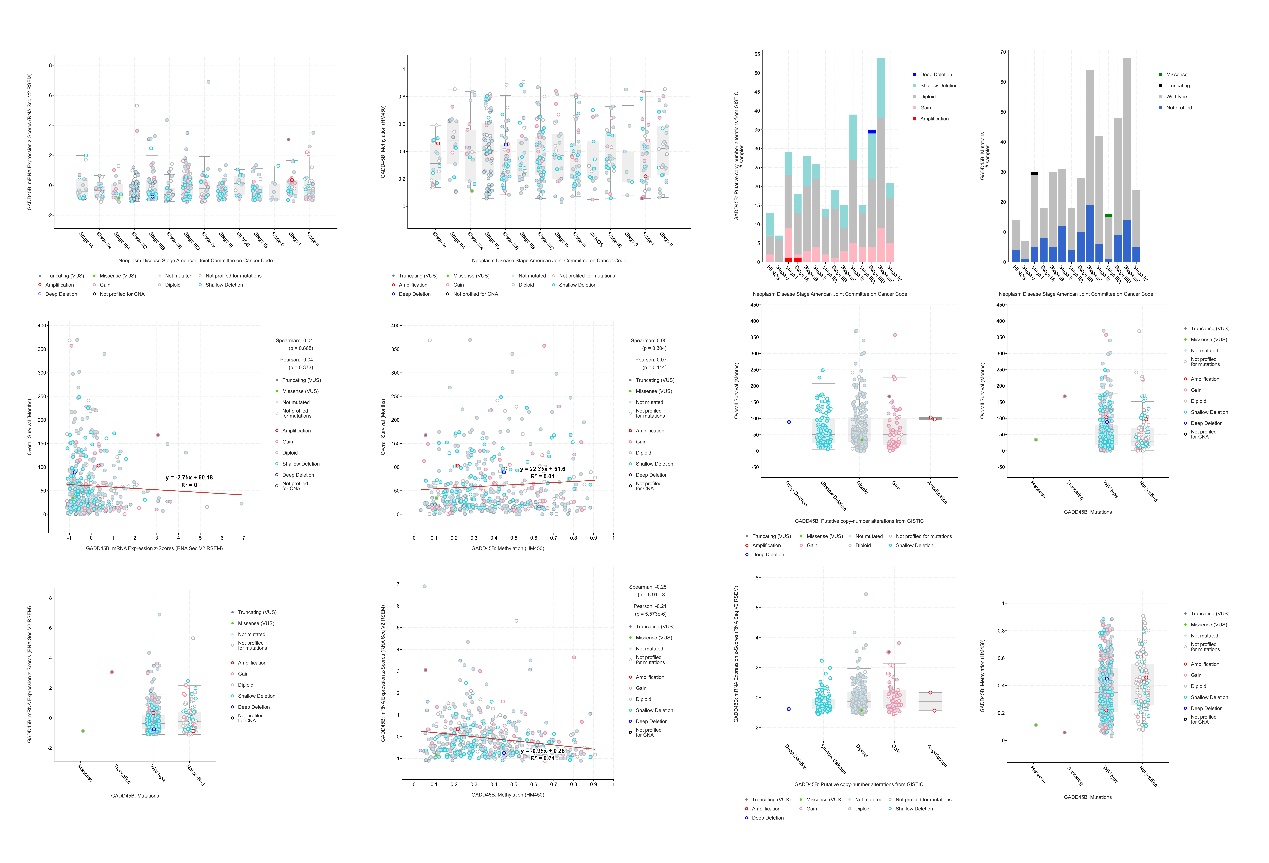


**Figure S13. The correlations of molecular feature and clinic data include neoplasm disease stage American joint committee on cancer code, overall survival, GADD45B mRNA expression, GADD45B mutation, GADD45B methylation, and GADD45B CN-alteration were showed with an individual legend.**


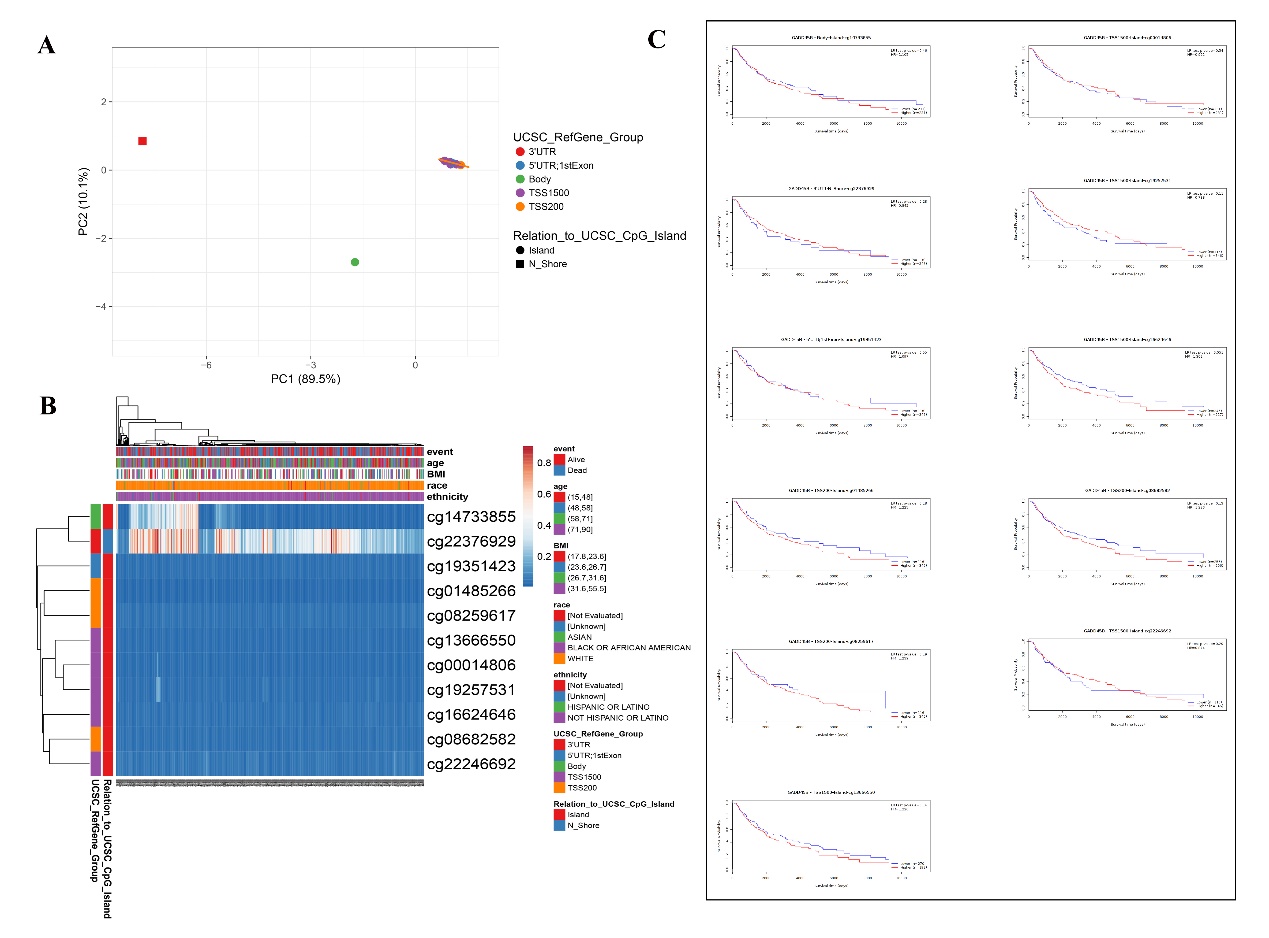


**Figure S14. Methylation analysis of GADD45B in melanoma. A,** Principal component analysis of samples and the legend on right. **B,** Heat map of cluster analysis for GADD45B methylation in melanoma patients and the legend on right. **C,** Kaplan meier plots of 11 types methylation in GADD45B.


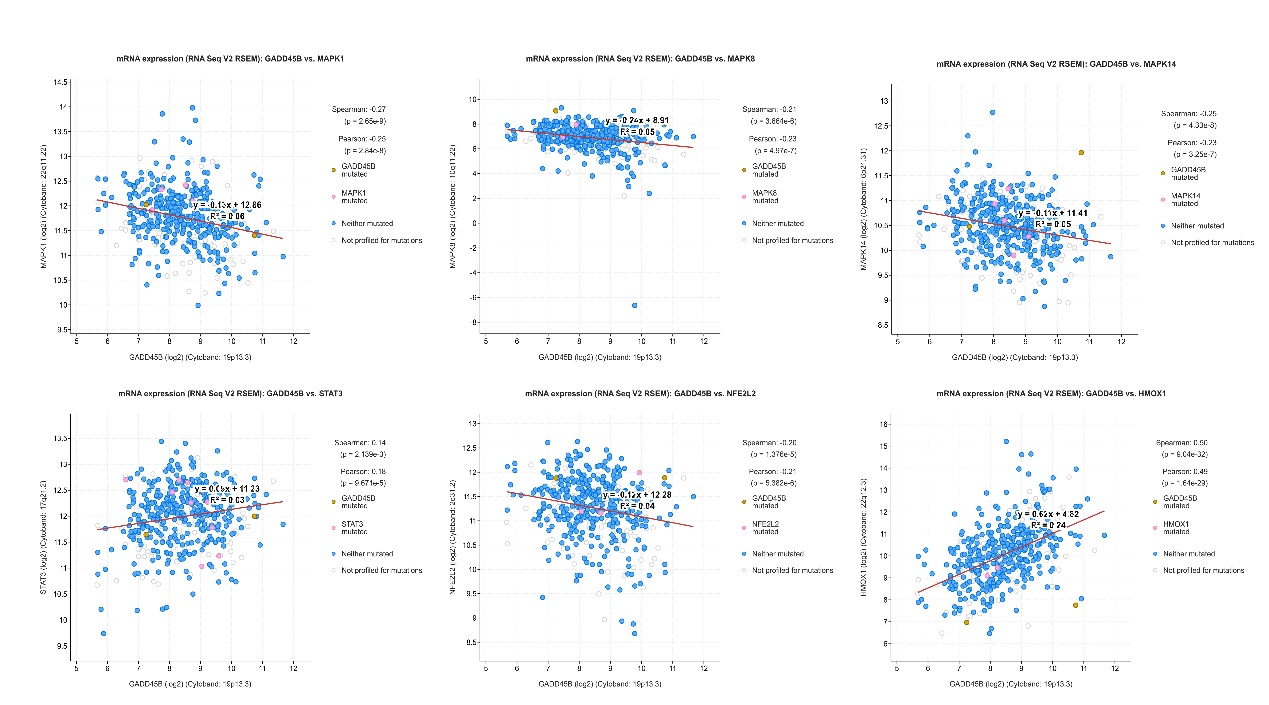


**Figure S15. Gene co-expression of GADD45B and MAPK1, MAPK8, MAPK14, STAT3, NFE2L2, HMOX1 were showed with an individual legend on right.** The red line represent the correlation of co-expression.

**
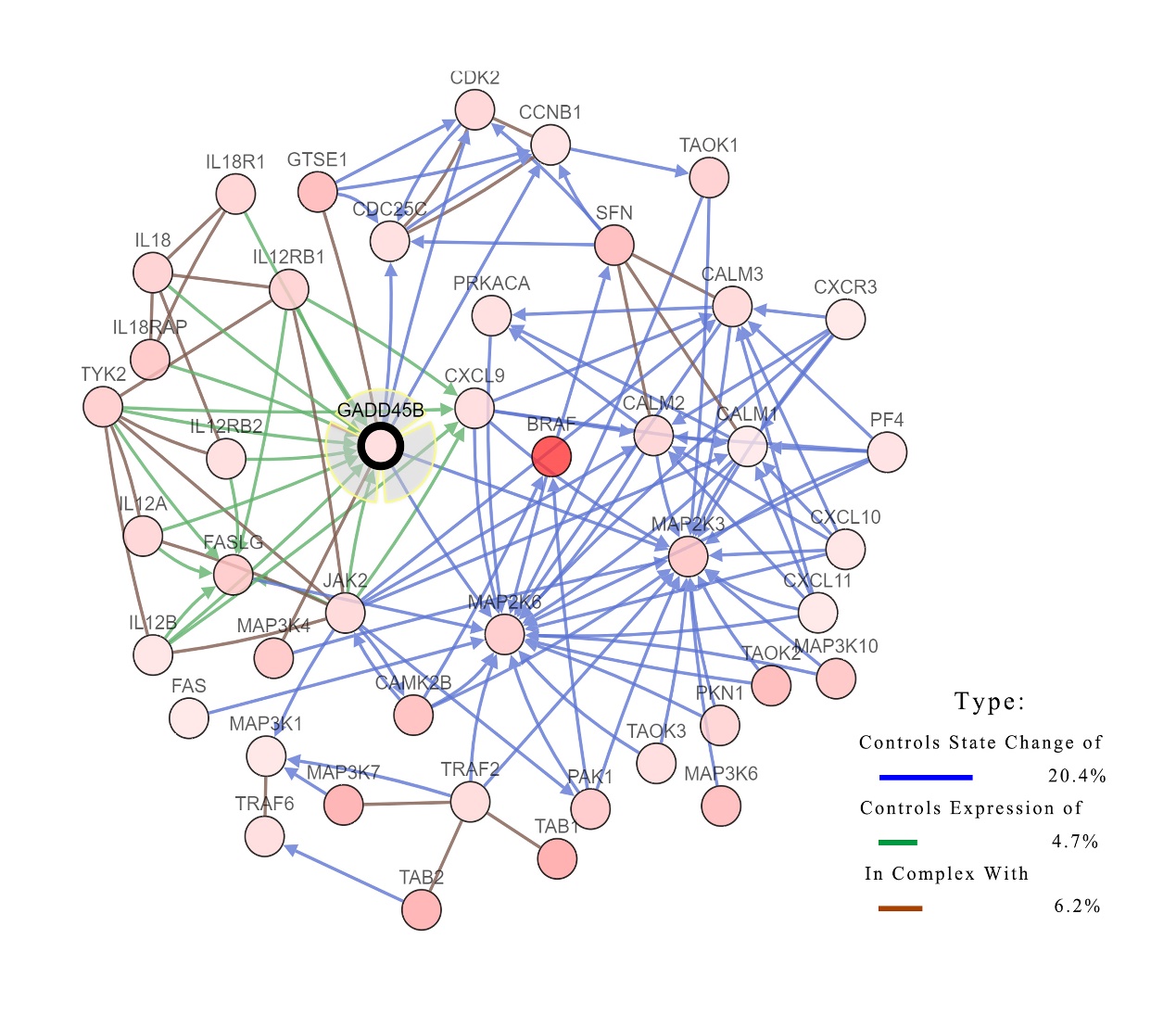
**

**Figure S16. Protein interaction network of GADD45B.** The colored lines represent different action relationships.

**
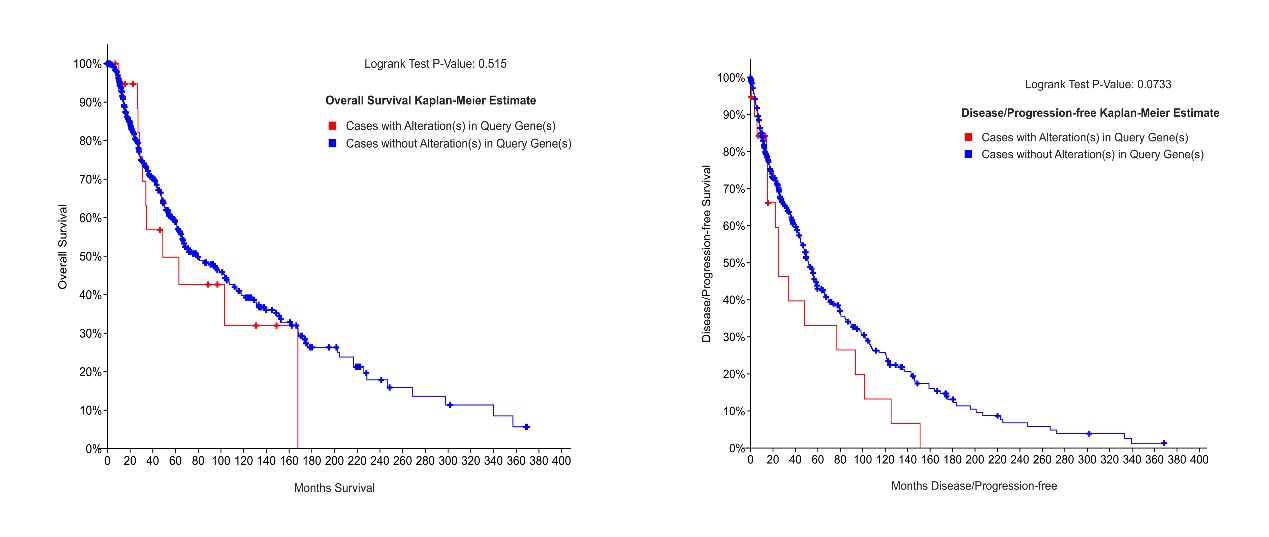
**

**Figure S17. Overall survival Kaplan-Meier estimate and disease/progression-free Kaplan-Meier estimate were showed respectively.** Red lines represent cases with alteration in GADD45B and blue lines represent cases without alteration.

**
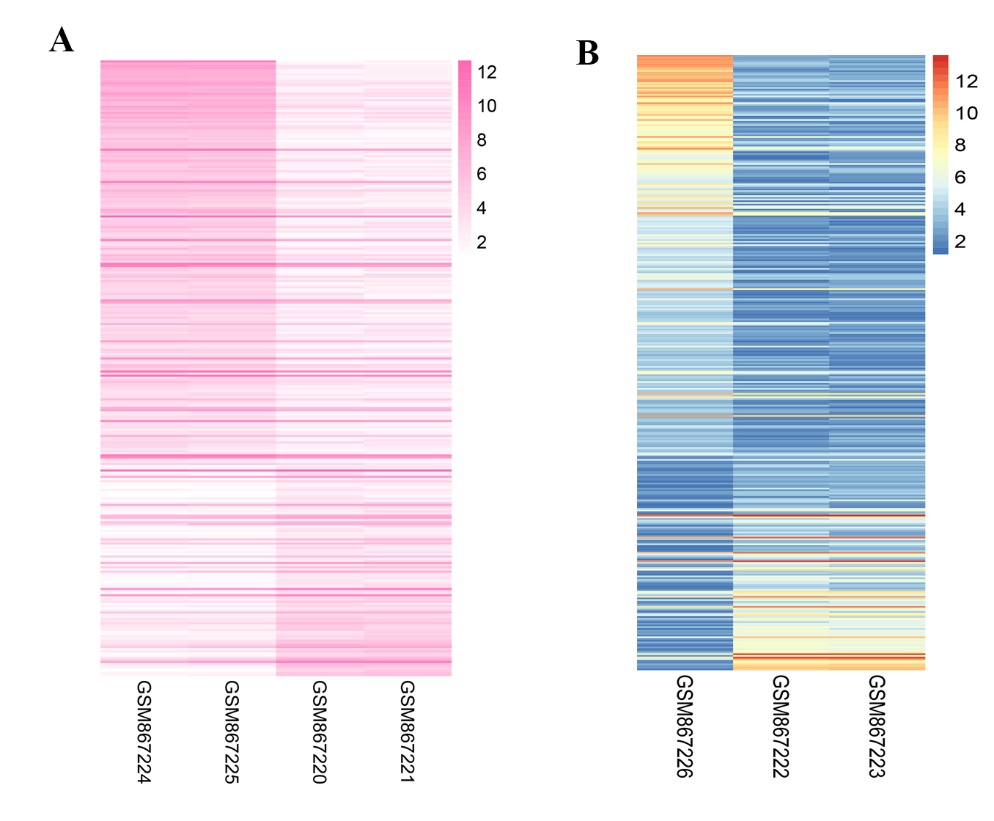
**

**Figure S18. A,** Heatmap of DEMs between normal melanocytes (GSM867224 and GSM867225) and melanoma (GSM867220 and GSM867221). **B,** Heatmap of DEMs comparing exosomes of normal melanocytes (GSM867226) and exosomes of melanoma (GSM867222 and GSM867223).

**
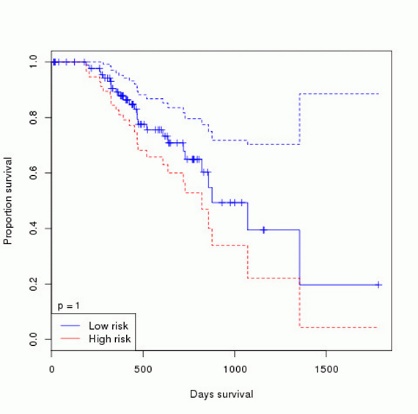
**

**Figure S19. Overall survival curve of miR-300.** Red lines represent cases with high risk and blue lines represent cases with low risk.

**
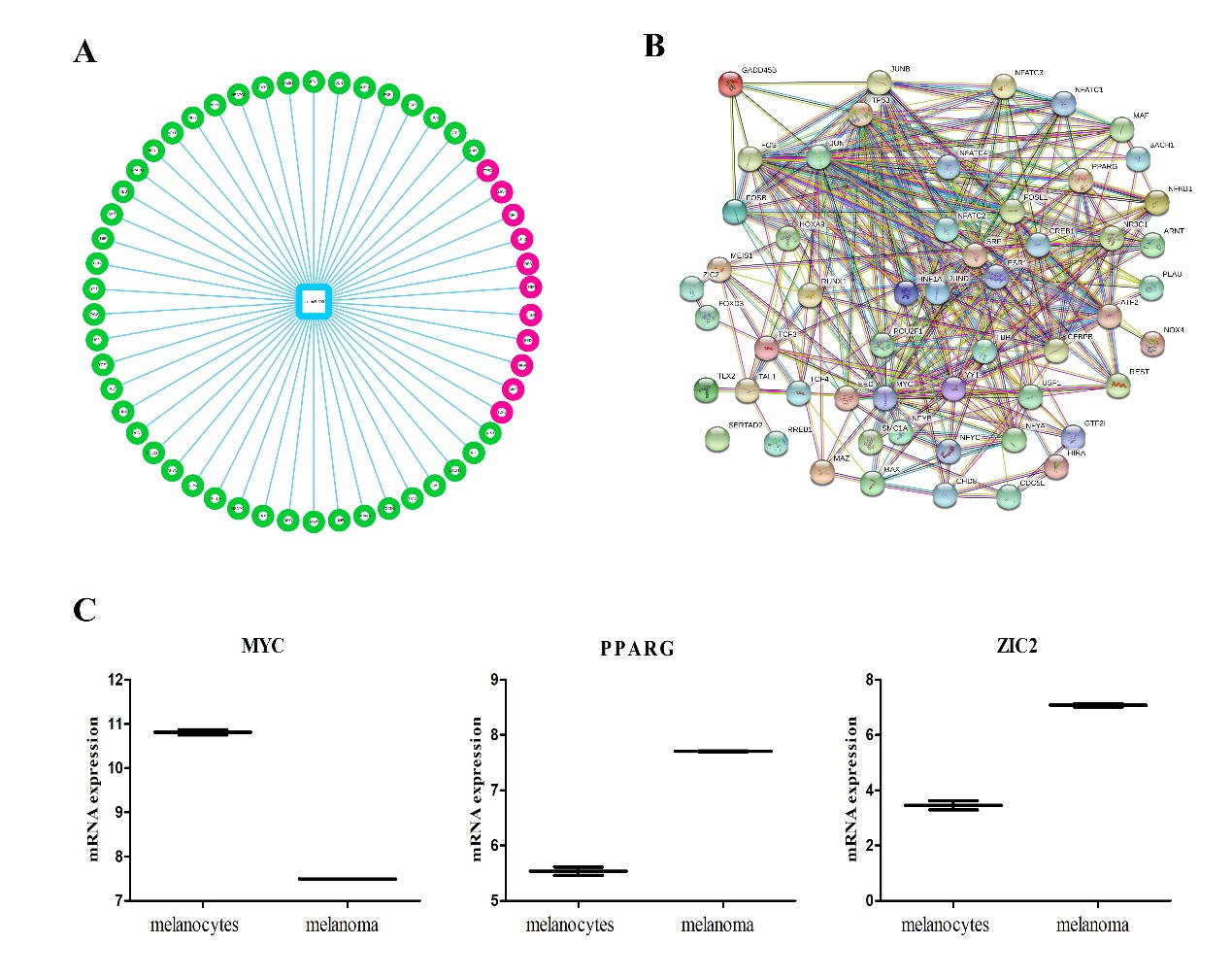
**

**Figure S20. A,** The predicted TFs of miR-300. The purple circles represent TFs dysregulated in melanoma or interact with GADD45B. The green circles represent undistinguished TFs. **B,** The interaction network of GADD45B and predicted TFs. **C,** Dysregulated TFs in melanoma were showed in box-plot.


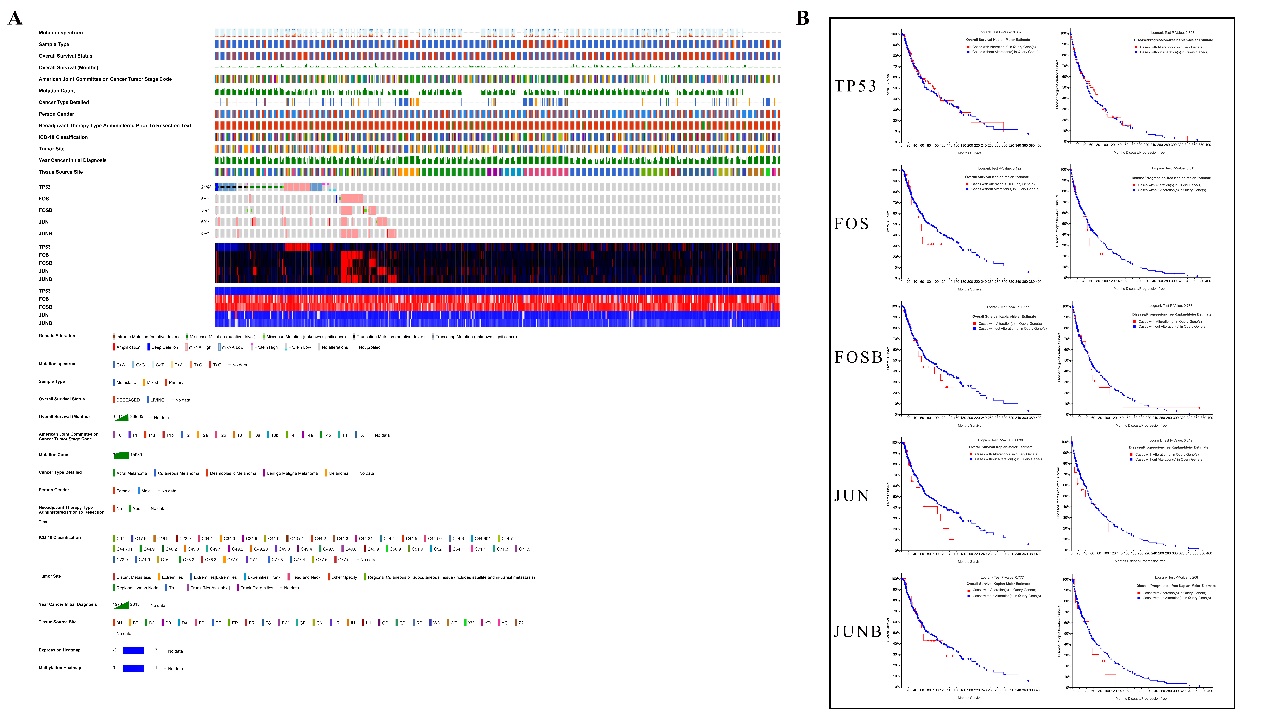


**Figure S21. TFs of miR-300 in melanoma based on TCGA. A,** Integrated plot of clinical data and TFs mutations in 472 melanoma samples. From top to bottom panels indicate: overall survival, American Joint Committee on Cancer tumor stage code, mutation count, cancer type detailed, person gender, neo-adjuvant therapy type administered prior to resection text, ICD-10 classification, tumor site, year cancer initial diagnosis, tissue source site, mutation symbols of TFs, heat map of TFs. The key to the color-coding is at the bottom. **B,** Overall survival Kaplan-Meier estimate and disease/progression-free Kaplan-Meier estimate were showed respectively. Red lines represent cases with alteration in specific TFs and blue lines represent cases without alteration.


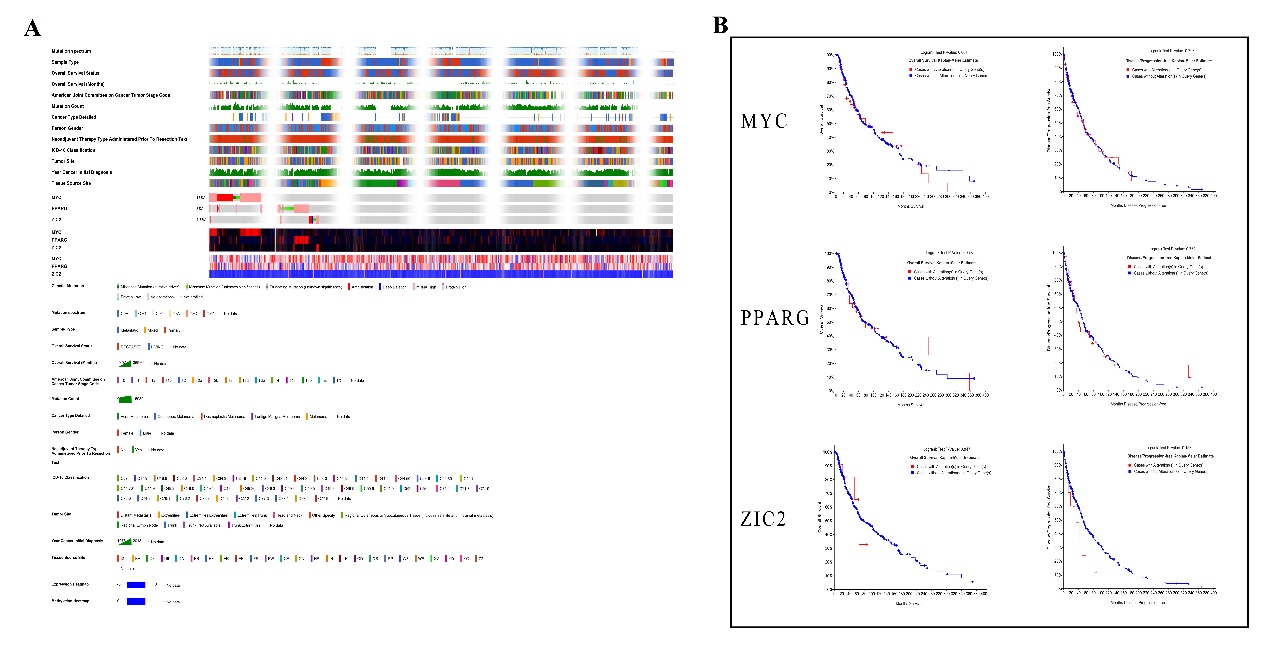


**Figure S22. TFs of miR-300 in melanoma based on TCGA. A,** Integrated plot of clinical data and TFs mutations in 472 melanoma samples. From top to bottom panels indicate: overall survival, American Joint Committee on Cancer tumor stage code, mutation count, cancer type detailed, person gender, neo-adjuvant therapy type administered prior to resection text, ICD-10 classification, tumor site, year cancer initial diagnosis, tissue source site, mutation symbols of TFs, heat map of TFs. The key to the color-coding is at the bottom. **B,** Overall survival Kaplan-Meier estimate and disease/progression-free Kaplan-Meier estimate were showed respectively. Red lines represent cases with alteration in specific TFs and blue lines represent cases without alteration.


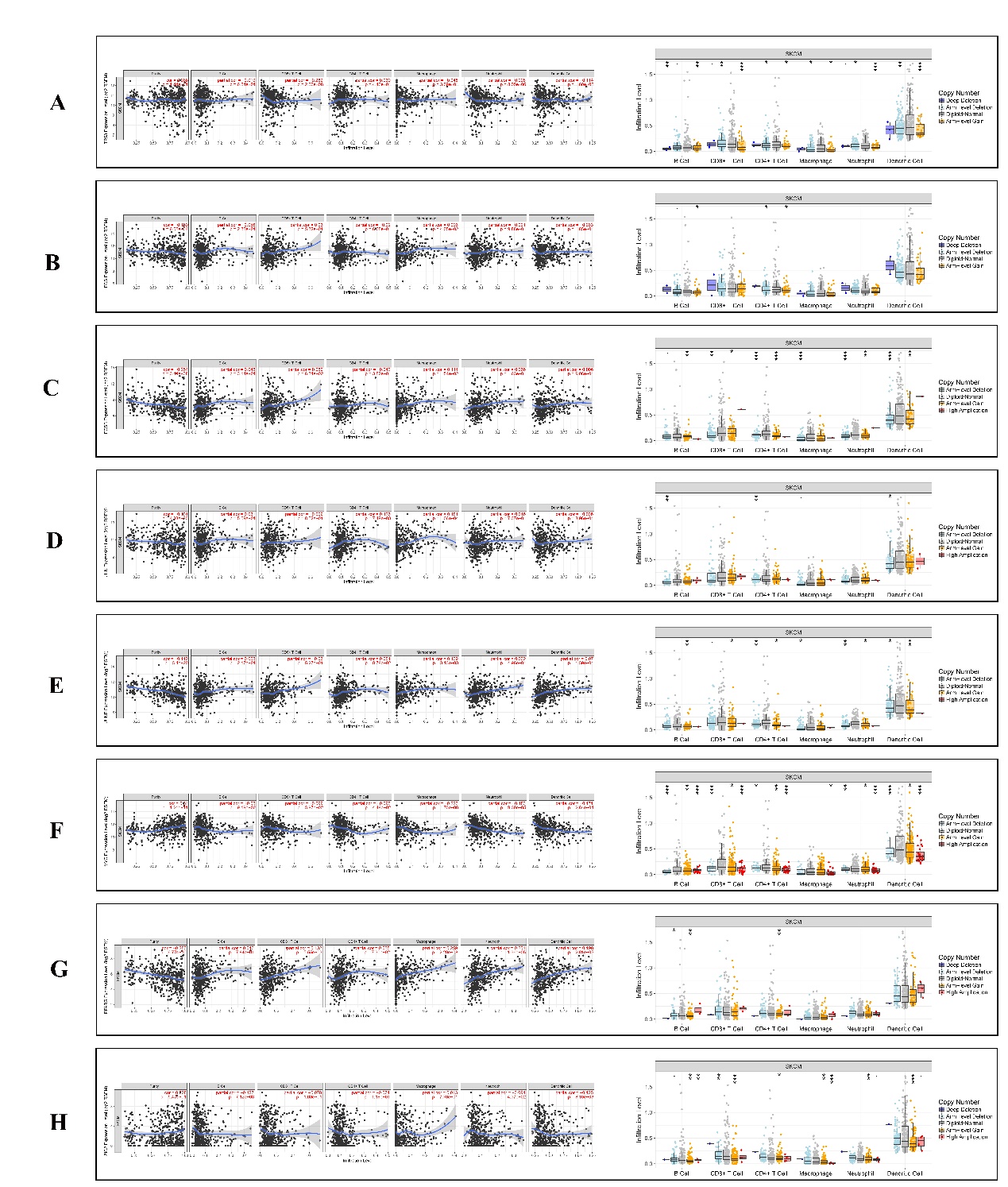


**Figure S23. The interaction of immune cell infiltration and specific factors. A-H,** The correlation of expression level vs. immune infiltration and CN vs. immune infiltration using two-sided Wilcoxon rank sum test


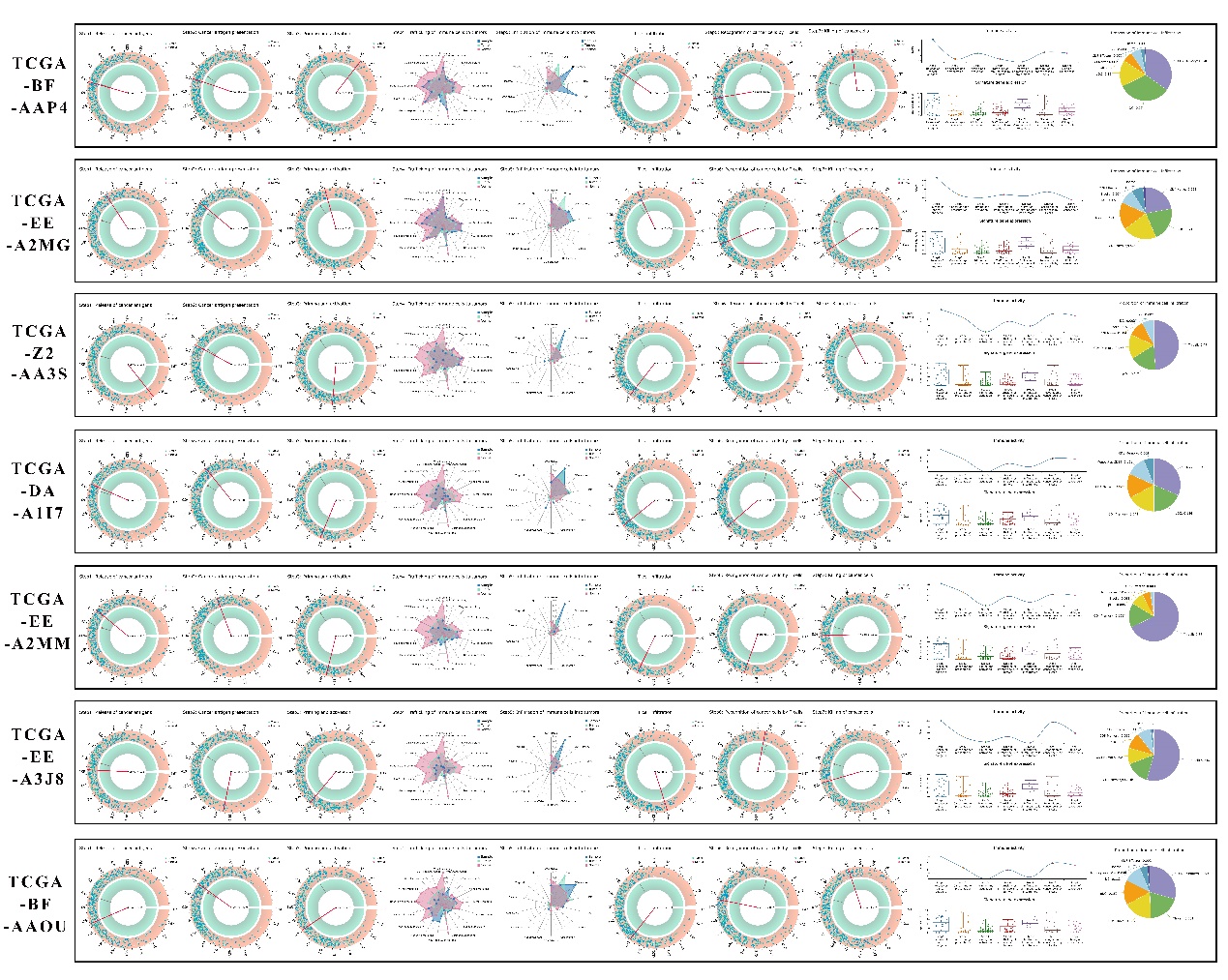


**Figure S24. Immunophenotype of patients with GADD45B alteration.** The immunity cycle of cancer was divided into 7 steps: 1, Release of cancer cell antigens. 2, Cancer antigen presentation. 3, Priming and activation. 4, Trafficking of immune cells to tumors. 5, Infiltration of immune cells into tumors. 6, Recognition of cancer cells by T cells. 7, Killing of cancer cells. From top to bottom are TCGA-BF-AAP4 (wild type), TCGA-EE-A2MG (amplification), TCGA-Z2-AA3S (amplification), TCGA-DA-A1I7 (deep deletion), TCGA-EE-A2MM (truncating mutation-Q79*),TCGA-EE-A3J8 (missense mutation-I89N) and TCGA-BF-AAOU (mRNA high). The scores of each step were calculated while immune activity, signature gene expression and immune cell infiltration were showed respectively.
